# Rare copy-number variants as modulators of common disease susceptibility

**DOI:** 10.1186/s13073-023-01265-5

**Published:** 2024-01-08

**Authors:** Chiara Auwerx, Maarja Jõeloo, Marie C. Sadler, Nicolò Tesio, Sven Ojavee, Charlie J. Clark, Reedik Mägi, Estonian Biobank Research Team, Tõnu Esko, Andres Metspalu, Lili Milani, Mari Nelis, Alexandre Reymond, Zoltán Kutalik

**Affiliations:** 1 Center for Integrative Genomics, University of Lausanne, Genopode building, 1015 Lausanne, Switzerland; 2 Department of Computational Biology, University of Lausanne, Genopode building, 1015 Lausanne, Switzerland; 3 Swiss Institute of Bioinformatics, 1015 Lausanne, Switzerland; 4 University Center for Primary Care and Public Health, 1005 Lausanne, Switzerland; 5 Institute of Molecular and Cell Biology, University of Tartu, 51010 Tartu, Estonia; 6 Estonian Genome Centre, Institute of Genomics, University of Tartu, 51010 Tartu, Estonia

**Keywords:** Structural variation, CNV, GWAS, Time-to-event analysis, Common diseases, Pleiotropy, 16p13.11, 16p11.2, Genomic disorders

## Abstract

**Background:**

Copy-number variations (CNVs) have been associated with rare and debilitating genomic disorders (GDs) but their impact on health later in life in the general population remains poorly described.

**Methods:**

Assessing four modes of CNV action, we performed genome-wide association scans (GWASs) between the copy-number of CNV-proxy probes and 60 curated ICD-10 based clinical diagnoses in 331,522 unrelated white British UK Biobank (UKBB) participants with replication in the Estonian Biobank.

**Results:**

We identified 73 signals involving 40 diseases, all of which indicating that CNVs increased disease risk and caused earlier onset. We estimated that 16% of these associations are indirect, acting by increasing body mass index (BMI). Signals mapped to 45 unique, non-overlapping regions, nine of which being linked to known GDs. Number and identity of genes affected by CNVs modulated their pathogenicity, with many associations being supported by colocalization with both common and rare single-nucleotide variant association signals. Dissection of association signals provided insights into the epidemiology of known gene-disease pairs (e.g., deletions in *BRCA1* and *LDLR* increased risk for ovarian cancer and ischemic heart disease, respectively), clarified dosage mechanisms of action (e.g., both increased and decreased dosage of 17q12 impacted renal health), and identified putative causal genes (e.g., *ABCC6* for kidney stones). Characterization of the pleiotropic pathological consequences of recurrent CNVs at 15q13, 16p13.11, 16p12.2, and 22q11.2 in adulthood indicated variable expressivity of these regions and the involvement of multiple genes. Finally, we show that while the total burden of rare CNVs—and especially deletions—strongly associated with disease risk, it only accounted for ~ 0.02% of the UKBB disease burden. These associations are mainly driven by CNVs at known GD CNV regions, whose pleiotropic effect on common diseases was broader than anticipated by our CNV-GWAS.

**Conclusions:**

Our results shed light on the prominent role of rare CNVs in determining common disease susceptibility within the general population and provide actionable insights for anticipating later-onset comorbidities in carriers of recurrent CNVs.

**Supplementary Information:**

The online version contains supplementary material available at 10.1186/s13073-023-01265-5.

## Background

Copy-number variants (CNVs) refer to duplicated or deleted DNA fragments (≥ 50 bp) and represent an important source of inter-individual genetic variation [1, 2]. As a highly diverse mutational class, CNVs can alter the copy-number of dosage-sensitive genes, induce gain- or loss-of-function (LoF) through gene fusion or truncation, unmask recessive alleles, or disrupt regulatory sequences, thereby representing potent phenotypic modifiers [3, 4]. As such, their role in human disease has mainly been studied in clinically ascertained cohorts, often presenting with congenital anomalies and/or severe neurological (e.g., developmental delay and intellectual disability or epilepsy) or psychiatric (e.g., autism or schizophrenia) symptoms [5–8]. Today, close to 100 genomic disorders (GDs), i.e., diseases caused by genomic rearrangements, have been described [9, 10]. Despite their deleteriousness, some of these CNVs are flanked by repeats and recurrently appear, remaining at a low but stable frequency in the population [11].

The emergence of large biobanks coupling genotype to phenotype data has fostered the study of CNVs in the general population. Whole genome sequencing represents the best approach to characterize the full human CNV landscape [1, 12, 13] but current long- and short-read sequencing association studies have a limited sample size [14–16]. Alternatively, larger sample sizes are available for exome sequencing data, offering the possibility to assess the phenotypic consequence of small CNVs [17–19], while microarray-based CNV calls are better-suited for the study of large CNVs and have been successfully used in association studies [9, 20–30]. Performing a CNV genome-wide association study (GWAS) on 57 medically relevant continuous traits in the UK Biobank (UKBB) [31], we previously identified 131 independent associations, including allelic series wherein carriers of CNVs at loci previously associated with rare Mendelian disorders exhibited subtle changes in disease-associated phenotypes but lacked the corresponding clinical diagnosis [27]. Paralleling findings for point mutations [32–35], this supports a model of variable expressivity, where CNVs can cause a wide spectrum of phenotypic alterations ranging from severe, early-onset diseases to mild subclinical symptoms, opening the question as to whether these loci are also associated with common diseases.

While continuous traits can be objectively measured in any individual, population cohorts, such as UKBB, have lower numbers of individuals with a disease compared to the population as a whole [36], leading to a case–control imbalance that reduces power compared to a balanced cohort of the same size. Moreover, defining cases relies on the dichotomization of complex underlying pathophysiological processes [37]. Beyond the inherent loss of power associated with the usage of binary variables [38], cases might be missed because an individual did not consult a physician, was misdiagnosed due to atypical clinical presentation, or is in a prodromal disease phase. Studies investigating CNV-disease associations in the general population have either focused on only a few diseases [29, 39–44] or well-established recurrent CNVs [24, 45–48]. Alternatively, high-throughput studies have assessed a broad range of continuous and binary traits simultaneously [18, 20, 21] without any precautions to accommodate the aforementioned challenges. To date, the largest disease CNV-GWAS meta-analyzed ~ 1,000,000 individuals [9]. While boosting power through increased sample size, it comes at the cost of extensive data harmonization, resulting in the exclusion of smaller CNVs (≤ 100 kb) and usage of broader disease categories (e.g., “immune abnormality”). Moreover, as this study includes several clinical cohorts, phenotypes are biased towards neuropsychiatric disorders (24/54 phenotypes) for which the role of CNVs is well-established [5–8].

Using tailored CNV-GWAS models mimicking four mechanisms of CNV action and time-to-event analysis, we investigate the relationship between CNVs and 60 carefully defined common diseases affecting a broad range of physiological systems in 331,522 unrelated white British UKBB participants. Extensively validating our results, we report associations according to confidence tiers and take advantage of rich individual-level phenotypic data to demonstrate the contribution of CNVs to the common disease burden in the general population.

## Methods

### Study material

#### Discovery cohort: UK Biobank

The UK Biobank (UKBB) is composed of ~ 500,000 volunteers (54% females) from the general UK population for which microarray-based genotyping and extensive phenotyping data—including hospital-based International Classification of Diseases, 10th Revision (ICD-10) codes (up to September 2021) and self-reported conditions—are available [31].

#### Replication cohort: Estonian Biobank

The Estonian Biobank (EstBB) is a population-based cohort of ~ 208,000 Estonian individuals (65% females; data freeze 2022v01 [12/04/2022]) for which microarray-based genotyping data and ICD-10 codes from crosslinking with national and hospital databases (up to end 2021) are available [49].

#### Software versions

CNVs were called with PennCNV v1.0.5 [50] using PennCNV-Affy (27/08/2009) and filtered based on a quality scoring pipeline [51]. Genetic analyses were conducted with PLINK v1.9 and v2.0 [52]. ANNOVAR (24/10/2019) was used to map genes to genetic regions [53]. Whenever genomic coordinates needed to be converted between builds, the UCSC Genome Browser LiftOver tool was used [54]. Statistical analyses were performed with R v3.6.1, and graphs were generated with R v4.1.3.

### CNV association studies in the UK Biobank

#### Microarray-based CNV calling

All results in this study are based on the human genome reference build GRCh37/hg19. UKBB genotype microarray data were acquired from two arrays with 95% probe overlap (Applied Biosystems UK Biobank Axiom Array: 438,427 samples; Applied Biosystems UK BiLEVE Axiom Array by Affymetrix: 49,950 samples) [31] and used to call CNVs as previously described [27]. Details about the CNV calling, quality control, and encoding in PLINK file sets are provided in Additional file 1: Note S1. Briefly, CNVs were called using standard PennCNV settings and samples with abnormal CNV profile were excluded. Remaining CNVs were attributed a probabilistic quality score (QS) that reflects the likelihood that the CNV call is a true positive [51]. The QS ranges from − 1 (likely deletion) to 1 (likely duplication), with intermediate values around 0 reflecting less confident CNV calls [51]. High-confidence CNVs, stringently defined by |QS|> 0.5, were retained and encoded in chromosome-wide probe-by-sample matrices with entries of 1, − 1, or 0 indicating probes overlapping a high-confidence duplication, deletion, or no/low-quality CNV, respectively. These matrices were converted into three PLINK binary file sets to accommodate association analysis according to four modes of CNV action.

#### Case–control definition and age of disease onset calculation

A pool of 331,522 unrelated white British UKBB participants (54% females) was considered after excluding related individuals (≤ 3rd degree), individuals with high genotype missingness (≥ 0.02), individuals that are not of white British ancestry (self-reported + genetically confirmed), CNV outlier samples based on genotyping plate or extreme CNV profile, and individuals reporting blood malignancies. Detailed criteria and number of individuals excluded at each step are described in Additional file 1: Note S2.

Cases and controls were defined for 60 ICD-10-based clinical diagnoses using *diagnosis – ICD10* (#41270), *cancer code, self-reported* (#20001), and *non-cancer illness code, self-reported* (#20002) to build exclusion and inclusion lists. For each disease, starting with the selected subset of 331,522 individuals previously described, we identified cases as individuals having received a specific, restricted set of ICD-10 codes matching our disease definition (i.e., inclusion list). We then defined our controls as individuals lacking both ICD-10 codes matching the case definition and self-reported or ICD-10 diagnoses of a broad set of conditions related to the assessed disorder (i.e., exclusion list). For instance, breast cancer controls should not have other cancers or radio-/chemotherapy, while schizophrenia controls should not have mood or personality disorders. For second-level ICD-10 codes, all subcodes are considered, otherwise only the specified ones. Finally, the disease burden was calculated as the number of diagnoses (out of the 60 assessed ones) an individual has received. For male- (prostate cancer) and female- (menstruation disorders, endometriosis, breast cancer, ovarian cancer) specific diseases, downstream analyses were conducted excluding individuals from the opposite sex.

Based on the *date at first in-patient diagnosis – ICD10* (#41280) and the individual’s *month* (#52) and *year* (#34) *of birth* (birthday assumed on average to be the 15th), the age at diagnosis was calculated by subtracting the earliest diagnosis date for codes on the inclusion list from the birth date and converting it to years by dividing by 365.25 to account for leap years.

#### Covariate and probe selection

To reduce computation time, relevant covariates and probes were pre-selected to fit tailored main CNV-GWAS models, with detailed methodology and quality controls reported in Additional file 1: Note S3. For each disease, a logistic regression was fitted to explain disease probability as a function of age (#21003), sex, genotyping array, and the 40 first principal components (PCs) from the single-nucleotide polymorphism (SNP) genotyping data. Nominally significantly associated covariates (*p* ≤ 0.05) were retained for the main analysis. Probe-level CNV, duplication, and deletion frequencies, i.e., the frequencies at which a probe is found to be overlapped by a CNV, duplication, or deletion, respectively, was estimated and probes with a CNV frequency < 0.01% were excluded. Retained probes were pruned at *r*^2^ > 0.9999 (--indep-pairwise 500 250 0.9999 PLINK v2.0) in the PLINK_CNV_ file set based on their CNV genotype. Two-by-three genotypic Fisher tests assessed dependence between disease status and probe copy-number (rows: control versus case; columns: deletion versus copy-neutral versus duplication; --model fisher PLINK v1.9; TEST column “GENO”) of the remaining probes. Fisher test *p*-values were not prone to strong genomic inflation. Finally, probes with *p*_Fisher_ ≤ 0.001 and a minimum of two disease cases among CNV, duplication, or deletion carriers were retained for assessment through the mirror/U-shaped, duplication-only, or deletion-only model, respectively.

#### Genome-wide significance threshold

Due to the recurrent nature of CNVs, the copy-number status of the 18,725 probes retained after frequency filter and pruning remain highly correlated and are thus not independent. Accounting for these 18,725 probes would result in an overly strict multiple testing correction. Using an established protocol [26, 27, 55], we estimated the chromosome-level number of effective tests and summed them up, resulting in an estimate of *N*_eff_ = 6,633, setting the genome-wide (GW) threshold for significance at *p* ≤ 0.05/6,633 = 7.5 × 10^−6^. This threshold is of the same order of magnitude as what others have estimated for disease CNV-GWASs [9]. We also assessed the number of associations surviving an experiment-wide threshold for significance that further accounts for the 60 assessed diseases (plus the disease burden), defined as *p* ≤ 0.05/(6,633*61) = 1.2 × 10^−7^. Enrichment for tier 1 and 2 associations (“Statistical confidence tiers”) among experiment-wide, as opposed to genome-wide, significant signals was assessed with a two-sided Fisher test.

#### Main CNV-GWAS model

Association between disease risk and copy-number of CNV-proxy probes was assessed through logistic regression with Firth fallback (--covar-variance-standardize --glm firth-fallback omit-ref no-x-sex hide-covar --ci 0.95 PLINK v2.0), using disease- and model-specific probes and covariates (“Covariate and probe selection”). Four association models were assessed: the mirror model assessed the additive effect of each additional copy (PLINK_CNV_ file set); the U-shape model assessed a consistent effect of any deviation from the copy-neutral state (PLINK_CNV_ file set, using the “hetonly” option in --glm PLINK v2.0); the duplication-only model (PLINK_DUP_ file set) assessed the impact of a duplication while disregarding deletions; the deletion-only model (PLINK_DEL_ file set) assessed the impact of a deletion while disregarding duplications. Effect sizes were harmonized to obtain the effect of the CNV—or of an additional copy for the mirror model—and the number of independent signals per disease was determined by stepwise conditional analysis (Additional file 1: Note S4). Briefly, for each disease and association model, the numerical CNV genotype of the lead probe was included along selected covariates in the logistic regression model and association studies were conducted anew in an iterative fashion until no probes passed the GW significant threshold. Characteristics of the most significant model (i.e., “best model”) are reported. The “main model” indicates which CNV type mainly drives the association, i.e., when associations were found through multiple models, priority was given to either the duplication-only or deletion-only models, otherwise to the model yielding the lowest *p*-value.

Due to the quantitative nature of the disease burden, the CNV-GWAS for that phenotype was based on linear regressions (--covar-variance-standardize --glm firth-fallback omit-ref no-x-sex hide-covar --ci 0.95 PLINK v2.0), correcting for selected covariates. Post-GWAS processing was performed as previously described [27].

#### CNV region definition and annotation

CNV region (CNVR) boundaries were defined by the most distant probes within ± 3 Mb and *r*^2^ ≥ 0.5 of each independent lead probe (--show-tags -tag-kb 3000 -tag-r2 0.5 PLINK v1.9; U-shape model: custom code). When multiple disease-CNV associations mapped to overlapping (≥ 1 bp) genomic coordinates, the CNVRs were merged, resulting in 45 unique, non-overlapping, disease-associated CNVRs, whose boundaries are defined as the maximal CNVR. Manual inspection ensured substantial overlap between merged CNVRs. CNVRs were annotated with hg19 HGNC and ENSEMBL gene names using annotate_variation.pl from ANNOVAR (--geneanno). Number of genes mapping to a CNVR was calculated and set to zero for CNVRs with REGION not equaling “exonic”.

#### Statistical confidence tiers

Following primary assessment through logistic regression (“Main CNV-GWAS model”), three statistical approaches were implemented to gauge robustness of the lead probe’s association signal. First, we assessed *post hoc* the *p*-value of the 2-by-3 genotypic Fisher tests (“Covariate and probe selection”). Second, we transformed the binary disease status into a continuous variable by computing the response residuals of the logistic regression of disease status on disease-relevant covariates. This allowed to use linear regressions to estimate the effect of the CNV genotype, encoded according to all significantly associated models in the primary analysis, on disease risk. The model generating the lowest *p*-value for the CNV encoding is reported. Third, time-to-event analysis was used to assess whether CNVs influence age of disease onset using Cox proportional hazards (CoxPH) models, the latter requiring an estimate for the age at last healthy measurement. For cases, age at last healthy measurement was defined as the age at disease diagnosis. For controls, age at last healthy measurement was defined by subtracting birth date from cutoff date (30/09/2021) and the resulting period was converted in years (“Case–control definition and age of disease onset calculation”). CoxPH models were fitted including disease-relevant covariates and numerically encoded CNV genotype for either of the four association models as predictors, using the coxph() function from the R survival package [56]. The model with the lowest CNV genotype *p*-value is reported. CNV-disease associations were classified in confidence tiers depending on whether they were confirmed by 3 (tier 1), 2 (tier 2), or 1 (tier 3) of the above-described approaches at the arbitrary validation significance threshold of *p* ≤ 1 × 10^−4^. Above-described validation strategies are not suitable for disease burden associations. As quantitative variables do not suffer from the same caveats as binary traits, we classified all disease burden associations as tier 1.

#### Literature-based supporting evidence

Using three literature-based approaches, we examined whether disease-associated CNVRs had previously been linked to relevant phenotypes. First, we investigated the colocalization of autosomal CNVRs with SNP-GWAS signals. GRCh38/hg38 lifted CNVR coordinates were inputted in the GWAS Catalog and associations (*p* ≤ 1 × 10^−7^) relevant to the investigated disease (i.e., disease itself, synonyms, continuous proxies, or major risk factors) were identified through manual curation. Second, we overlapped OMIM morbid genes (i.e., linked to an OMIM disorder; morbidmap.txt) with disease-associated CNVRs. Through manual curation, we flagged OMIM genes associated with Mendelian disorders sharing clinical features with the common disease associated through CNV-GWAS. Third, we examined if implicated CNVRs overlapped regions at which CNVs were found to modulate continuous traits [27] or disease risk [21, 24].

### Replication in the Estonian Biobank

Disease cases and disease burden in the EstBB were defined using the same inclusion and exclusion criteria as for UKBB, with exceptions described in Additional file 1: Note S5. Autosomal CNVs were called from Illumina Global Screening Array genotype data for 193,844 individuals of European ancestry that survived general quality control and CNV-specific quality control as described in Additional file 1: Note S5. High-confidence CNV calls (|QS|> 0.5) of the 156,254 remaining individuals were encoded into three PLINK binary file sets (Additional file 1: Note S1). Related individuals with available CNV calls were pruned (< 3rd degree relatedness), leaving 90,211 unrelated samples for the replication study. Disease-relevant covariates were selected among sex, year of birth, genotyping batch (1–11), and PC1-20. EstBB probes overlapping the 68 UKBB autosomal disease-associated CNVRs were filtered for a CNV, duplication, or deletion frequency ≥ 0.01% and were tested for association according to the mirror/U-shape, duplication-only, or deletion-only model, respectively, depending on the best UKBB model. Summary statistics of the EstBB probe with the closest genomic location to the lead UKBB probe whose regression did not fail could be retrieved for 49 signals, setting the replication threshold for significance at *p* ≤ 0.05/49 = 1.0 × 10^−3^. *P*-values were adjusted to account for directional concordance with UKBB effects by rewarding (${p}_{new}= \frac{{p}_{old}}{2}$) and penalizing (${p}_{new}= 1-\frac{{p}_{old}}{2}$) signals with matching and non-matching effect size signs, respectively. One-sided binomial tests (binom.test()) were used to assess enrichment of observed versus expected significant replications at various thresholds ($\alpha$= 0.1 to 0.005 by steps of 0.005). Details of the replication analysis are in Additional file 1: Note S5.

### BMI confounding analysis

We sought to assess whether some of our associations might be driven by the CNVR’s effect on body mass index (BMI). Average BMI (#21001) over available instances was used. For an association to be tested for possible confounding, we required that (i) BMI significantly associated with disease risk (*p* ≤ 0.05/61 = 8.2 × 10^−4^) in a model including all disease-specific covariates and (ii) the CNV genotype of the lead probe encoded numerically according to the best model to significantly associate with BMI (*p* ≤ 0.05/73 = 6.8 × 10^−4^) previously inverse normal transformed and corrected for age, age^2^, sex, genotyping batch, and PC1-40. Twenty-five association signals matched these criteria and for them, we fitted a logistic regression (or linear regression for the disease burden) with disease status as outcome and lead probe encoded numerically according to the best model, disease-specific covariates, and BMI as predictors. Significant differences in CNV effect sizes upon BMI adjustment were assessed by two-sided *t*-test and deemed significant at *p* ≤ 0.05/25 = 0.002. Associations likely driven by BMI were defined as those for which the CNV effect *p*-value dropped below the GW significance threshold upon adjustment for BMI.

### CNV region constraint analysis

Evolutionary constraint of genes overlapping disease-associated CNVRs, i.e., “disease genes,” was assessed by comparing their probability of LoF intolerance (pLI), loss of function observed/expected upper bound fraction (LOEUF), probability of haploinsufficiency (pHaplo), and probability of triplosensitivity (pTriplo) scores to the ones of “background genes” with a two-sided Wilcoxon rank-sum test. Background genes were identified by annotating ranges of one or multiple consecutive probes with CNV frequency ≥ 0.01% with ANNOVAR (hg19 HGNC gene names) and excluding disease genes. For pLI and LOEUF, all disease genes were considered together. For pHaplo and pTriplo, two disease gene groups were considered: genes overlapping CNVRs with at least one association through the duplication-only model and genes overlapping CNVRs with at least one association through the deletion-only model. As many CNVRs associated through both models, the analysis was repeated considering genes overlapping CNVRs with at least one association through the duplication-only and none through the deletion-only model and vice versa.

### Extended phenotypic assessment

#### 17q12 deletion

For time-to-event analysis, the same chronic kidney disease (CKD) definition as in the main analysis was used. Low-quality CNVs (|QS|≤ 0.5) were excluded from analyses. Time-to-event analysis was performed as previously described (“Statistical confidence tiers”), modeling both 17q12 deletions and duplications in the same CoxPH model adjusted for sex, age, age^2^, array, and PC1-40. Estimated glomerular filtration rate (eGFR) was calculated based on the CKD-EPI equation using #30700 (*creatinine* [µmol/L]), accounting for age, sex, and ancestry [57].

#### Subgrouping of CNV carriers

For fine-mapping of association signals, CNV carriers were divided into subgroups based on visual inspection of CNV breakpoints (BPs) and segmental duplications. Used coordinates are in Additional file 1: Note S6.

#### CNV versus copy-neutral comparisons

Comparisons between groups of CNV carriers and copy-neutral individuals always exclude low-quality CNV (|QS|≤ 0.5) carriers altogether. For diseases, prevalence is estimated as $\mathrm{q}=\frac{\mathrm{c}}{\mathrm{n}}$ , with $\mathrm{c}$ and $\mathrm{n}$ representing the number of cases and total number of individuals in a group, and $\mathrm{SE}(\mathrm{q})= \sqrt{\frac{\mathrm{q}*(1-\mathrm{q})}{\mathrm{n}}}$. Differences in prevalence compared to copy-neutral individuals were assessed with a two-sided Fisher test. For continuous traits, comparisons were based on two-sided *t*-tests.

### CNV burden analyses

#### CNV burden association studies

In the UKBB, individual-level CNV, duplication, and deletion burden were calculated as the number of Mb or genes affected by high-confidence (|QS|> 0.5) autosomal CNVs, duplications, and deletions, respectively, yielding six CNV burden metrices, as previously described [27]. Variance explained by these six CNV burden metrices was estimated by fitting logistic or linear regressions predicting disease outcome or disease burden as a function of the CNV burden metric (without any covariates) and assessing the McFadden pseudo-R^2^ or the adjusted R^2^ of the regression, respectively. Association between the six CNV burden metrices and the 60 diseases (logistic regression) or the disease burden (linear regression) were assessed including disease-relevant covariates in the model. Accounting for the 61 evaluated traits, significance was defined at *p* ≤ 0.05/61 = 8.2 × 10^−4^.

#### CNV burden association studies corrected for CNV-GWAS signals

For each disease, CNVs, duplications, and deletions overlapping (≥ 1 bp) a CNVR significantly associated with the disease of interest through CNV-GWAS were omitted from the CNV, duplication, and deletion burden calculations if the CNVR had been found to associate with the disease through the mirror/U-shape, duplication-only, or deletion-only model, respectively. Association studies were repeated as previously described using corrected burden values.

#### Partitioned CNV burden association studies

To determine which part of the genome was driving the associations between disease risk and the CNV burden, we defined 5 genomic partitions:

- CNVR partition: 40 autosomal disease-associated CNVRs identified in this study. CNVRs were considered for the CNV, duplication, and deletion burden, except for CNVRs yielding associations uniquely through the duplication-only or deletion-only models, which were considered only for the duplication and deletion burdens.
- GD partition: 92 GDs curated by Crawford *et al*. [24]. Duplication syndromes were considered for the duplication burden, deletion syndromes for the deletion burden, and all genomic disorders were considered for the CNV burden.
- R1 partition: Intersect between the CNVR and GD partitions, encompassing nine disease-associated CNVRs and 20 GDs caused by 10 reciprocal CNVs.
- R2 partition: 72 GDs not included in the R1 partition.
- R3 partition: 31 autosomal CNVRs not included in the R1 partition.

For every individual, we identified and summed up the subset of CNVs, duplications, and deletions (measured in number of genes or number of Mb) that overlaps these partitions (i.e., “subset burden”). Overlaps were defined either as (i) any overlap (≥ 1 bp) with the regions defined by the partition, or more stringently, (ii) by reciprocal 50% bp overlap (i.e., the CNV covers > 50% of the partition’s region and the partition’s region covers > 50% of the CNV). The subset burden was subtracted from the total burden (i.e., “corrected burden”). Association studies were repeated as previously described using subset and corrected burden metrices.

## Results

### The spectrum of common diseases in the UK Biobank

**Fig. 1:**
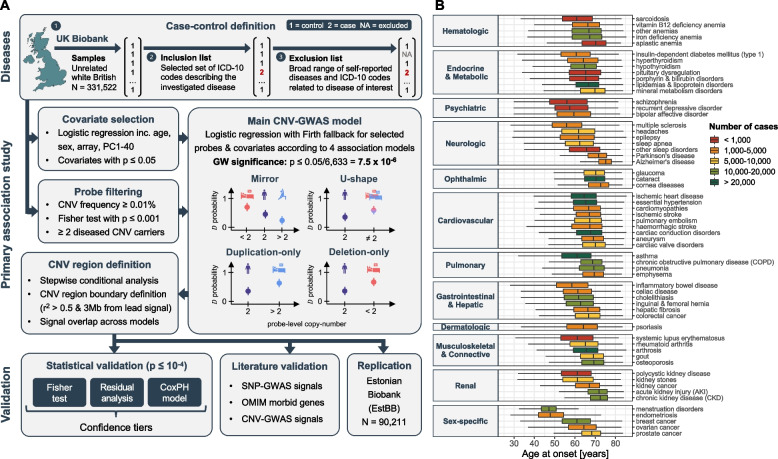
Overview of the study. **A** Schematic representation of the analysis workflow. Diseases: For each of the 60 investigated diseases, 331,552 unrelated white British individuals were divided into three subsets: controls (encoded as 1; step 1), cases with the disease (encoded as 2; step 2), and a subset of individuals who were excluded because they had conditions similar but not identical to the disease (encoded as NA; step 3). Primary association study: Disease-specific relevant covariates were selected. Probes were pre-filtered based on copy-number variant (CNV) frequency, Fisher test association *p*-value, and presence of ≥ 2 diseased carriers. Disease- and model-specific covariates and probes were used to generate tailored genome-wide CNV association scans (CNV-GWASs) based on Firth fallback logistic regression according to a mirror, U-shape, duplication-only (i.e., considering only duplications), and deletion-only (i.e., considering only deletions) models. Independent lead signals were identified through stepwise conditional analysis and CNV regions were defined based on probe correlation and merged across models. Validation: Statistical validation methods (i.e., Fisher test, residuals regression, and Cox proportional hazards model (CoxPH)) were used to rank associations in confidence tiers. Literature validation approaches leverage data from independent studies to corroborate that genetic perturbation (single-nucleotide polymorphisms (SNP), rare variants from the OMIM database, or CNVs) in the region are linked to the disease. Independent replication in the Estonian Biobank. **B** Age of onset for the 60 assessed diseases, grouped based on ICD-10 chapters and colored according to case count. Data are represented as boxplots; outliers are not shown

Sixty disorders spanning 12 ICD-10 chapters were selected to cover a wide range of physiological systems, favoring conditions with sufficiently large sample size and a likely genetic basis (Fig. 1; Additional file 2: Figure S1; Additional file 3: Table S1). We used a three-step approach to designate cases and controls in the UKBB (Fig. 1A; top): Starting from 331,522 unrelated white British individuals, we defined cases based on a narrow list of hospital-based diagnoses (i.e., ICD-10 codes) and excluded self-reported cases, as well as self-reported and hospital diagnoses of related conditions from controls. Except for systemic lupus erythematosus (*N* = 422) and polycystic kidney disease (*N* = 454), all diseases had over 500 cases. Nineteen diseases had a case count > 10,000, with arthrosis (*N* = 62,175) and essential hypertension (*N* = 97,860) being the most frequent. Seven diseases had a median age of onset ≤ 60 years, predominantly female reproductive disorders, autoimmune conditions, and psychiatric diseases. Conversely, the nine diseases with a median age of onset ≥ 70 years were mainly degenerative disorders of the brain, eye, and kidney, overall aligning with epidemiological knowledge of the respective diseases (Fig. 1B).

### Copy-number variant genome-wide association study

To assess whether disease susceptibility is modulated by CNVs, we performed CNV-GWASs, i.e., test if the copy-number of CNV-proxy probes influences the probability to develop a disease or an individual’s disease burden (Fig. 1A; middle). Briefly, microarray-called CNVs for 331,522 unrelated white British individuals were transformed to the probe level after quality control [27]. To further reduce the number and complexity of implemented logistic regressions, pre-processing steps selected relevant covariates and probes for each disease and model combination, thereby lowering computation time (Additional file 1: Note S3; Additional file 3: Tables S2-3). As CNVs can act through different gene dosage mechanisms, four association models were assessed: mirror and U-shape models consider deletions and duplications simultaneously, assuming that they impact disease risk in opposite or identical direction, respectively, while the CNV type-specific duplication- and deletion-only models assess independently the effect of duplications and deletions, respectively. All summary statistics are publicly available.

**Fig. 2:**
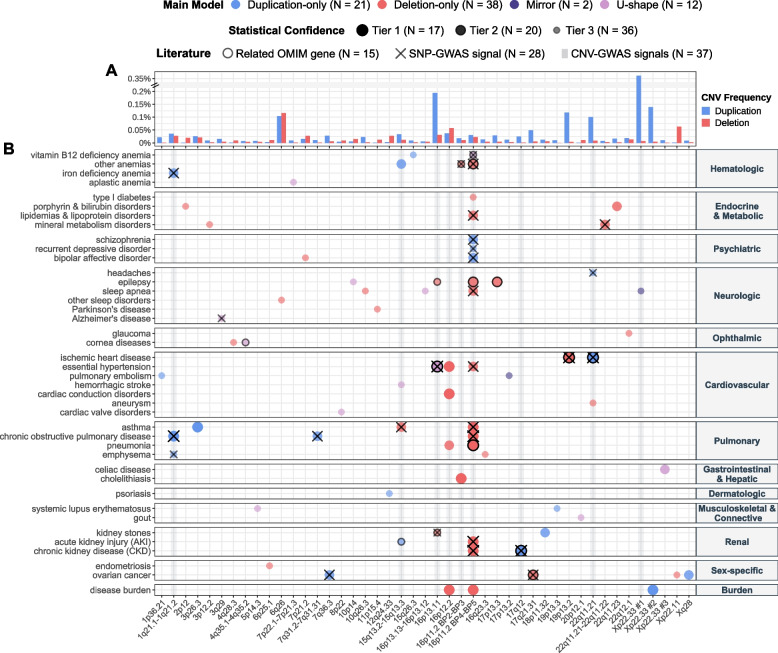
CNV-disease association map. **A** Duplication and deletion frequencies ([%]; *y*-axis; break: //) of the lead probe for each unique and non-overlapping disease-associated CNV region (CNVR), labeled with corresponding cytogenic band (*x*-axis; 16p11.2 is split to distinguish the distal 220 kb BP2-3 and proximal 600 kb BP4-5 CNVRs; non-overlapping CNVRs on the same cytogenic band are numbered). If signals mapping to the same CNVR have different lead probes, the maximal frequency was plotted. **B** Associations between CNVRs (*x*-axis) and diseases (*y*-axis) identified through CNV-GWAS. Color indicates the main association model. Size and transparency reflect the statistical confidence tier. Black contours indicate overlap with OMIM gene causing a disease with shared phenotypic features. Black crosses indicate overlap with SNP-GWAS signal for a related trait. Gray shaded vertical lines indicate CNVRs with continuous trait associations [27]. *N* provides count for various features

Stepwise conditional analysis narrowed GW significant associations (*p* ≤ 7.5 × 10^−6^; see “Methods” for threshold calculation) to 40, 41, 21, and 38 independent signals for the mirror, U-shape, duplication-only, and deletion-only models, respectively. These were combined into 70 risk-increasing (i.e., no disease-protecting CNV) disease associations and 3 disease burden associations that map to 45 unique, non-overlapping, disease-associated CNVRs (Fig. 2; Table 1; Additional file 3: Table S4), among which nine (20%) could be unambiguously linked to a known GD. Forty-five associations (45/73 = 62%) were supported at GW significance by multiple models, the lowest *p*-value (i.e., “best model”) being obtained through the mirror, deletion-only, U-shape, and duplication-only models for 24, 23, 21, and 5 of the signals, respectively. No association was detected at GW significance by both the duplication-only and deletion-only models, so that each signal was attributed a “main model” that indicates whether the association is primarily driven by duplications or deletions (Fig. 2). The main model should be interpreted with caution as both deletions and duplications might influence disease risk but only one CNV type-specific model might reach GW significance, due for instance to higher frequency of one CNV type. This is particularly relevant as 73% of the 45 disease-associated CNVRs have a higher duplication than deletion frequency (Fig. 2A). Hence, 20 out of 21 (95%) of signals mainly driven by duplications were also identified by the mirror/U-shape model(s) and contribution of deletions cannot be excluded.

**Table 1:** 45 disease-associated CNV regions. Cytogenic band and genomic coordinates (GRCh37/hg19) of the 45 unique, non-overlapping, disease-associated CNV regions (CNVRs) depicted on the *x*-axis of Fig. 2. For each CNVR, length in kilobase pairs is given (“Size”). “GD” indicates whether the CNVR matches any of the 92 genomic disorders (GD) compiled by Crawford *et al*. [24]: Y = “yes”; * = partial overlap with the 22q11.2 distal CNVR (chr22:21,920,000–23,650,000). Disease associations mapping to that CNVR are listed, with bold font indicating that the association is likely mediated by increased body mass index (BMI). All the models through which the association was detected at genome-wide significance are indicated in superscript: “U” = U-shape model; “ + ” = duplication-only model; “ − ” = deletion-only model; “M” = mirror model; CKD = chronic kidney disease; AKI = acute kidney injury

**Cytogenic band**	**Chr**	**CNVR start**	**CNVR end**	**Size [kb]**	**GD**	**Disease associations**
**1p36.21**	1	12,854,105	13,038,285	184		Pulmonary embolism^U,+,M^
**1q21.1-1q21.2**	1	146,478,785	147,832,715	1,354	Y	Chronic obstructive pulmonary disease^U,+^, emphysema^+,M^, iron deficiency anemia^U,+^
**2p12**	2	78,376,475	78,680,202	304		Disorders of bilirubin metabolism^U, −,M^
**3p26.3**	3	2,141,411	2,465,091	324		Asthma^+,M^
**3p12.2**	3	80,344,634	83,400,564	3,056		Disorders of mineral metabolism^−,M^
**3q29**	3	196,953,177	197,331,898	379	Y	Alzheimer’s disease^U^
**4q28.3**	4	136,510,759	136,952,267	442		Cornea diseases^U, −,M^
**4q35.1-4q35.2**	4	186,687,554	187,182,384	495		Cornea diseases^U^
**5p14.3**	5	20,254,182	20,924,403	670		Systemic lupus erythematosus^U^
**6p25.1**	6	4,235,784	4,658,277	422		Endometriosis^−,M^
**6q26**	6	162,705,164	162,873,489	168		Sleep disorders^−,M^
**7p22.1-7p21.3**	7	7,260,027	7,504,011	244		Aplastic anemia^U^
**7p21.2**	7	15,074,783	15,249,515	175		Bipolar disorder^−,M^
**7q31.2-7q31.31**	7	117,399,981	119,333,169	1,933		Chronic obstructive pulmonary disease^U,+,M^
**7q36.3**	7	158,530,132	158,953,160	423		Ovarian cancer^U,+,M^
**8p22**	8	17,599,136	17,719,930	121		Cardiac valve disorders^U^
**10p14**	10	6,677,540	6,833,390	156		Epilepsy^U^
**10q26.3**	10	135,217,002	135,237,176	20		Sleep apnea^−^
**11p15.4**	11	5,322,902	5,417,034	94		Parkinson’s disease^U, −,M^
**12q24.33**	12	131,611,538	131,825,359	214		Psoriasis^+,M^
**15q13.2-15q13.3**	15	30,912,719	32,516,949	1,604	Y	AKI^+^, anemia^U,+^, asthma^−,M^, **hemorrhagic stroke**^U^
**15q26.3**	15	101,319,208	101,613,151	294		Vitamin B12 anemia^U,+,M^
**16p13.13-16p13.12**	16	12,516,765	12,659,427	143		Sleep apnea^U^
**16p13.11**	16	15,120,501	16,353,166	1,233	Y	Epilepsy^−^, hypertension^U^, kidney stones^−^
**16p12.2**	16	21,946,523	22,440,319	494	Y	Cardiac conduction disorders^U, −,M^, disease burden^U, −,M^, hypertension^−,M^, pneumonia^−^,
**16p11.2 BP2-BP3**	16	28,775,159	29,043,450	268	Y	**Anemia**^−^, **cholelithiasis**^−,M^
**16p11.2 BP4-BP5**	16	29,596,230	30,208,637	612	Y	AKI^U, −,M^, **anemia**^U, −^, **asthma**^−^, bipolar disorder^U,+,M^, chronic obstructive pulmonary disease^U, −,M^, CKD^U, −^, disease burden^U, −,M^, **epilepsy**^−^, **hypertension**^−^, **lipidemias and lipoprotein disorders**^−^, pneumonia^U, −,M^, recurrent depressive disorder^U,+,M^, schizophrenia^U,+,M^, **sleep apnea**^−,M^, **type I diabetes**^−^, vitamin B12 anemia^U^
**16q23.3**	16	82,954,230	83,133,760	180		Emphysema^−^
**17p13.3**	17	631,380	738,187	107		Epilepsy^−,M^
**17p13.2**	17	4,378,105	4,498,641	121		Pulmonary embolism^M^
**17q12**	17	34,755,219	36,249,489	1,494	Y	CKD^U,+,M^
**17q21.31**	17	41,197,733	41,276,111	78		Ovarian cancer^−,M^
**18p11.32**	18	685,968	1,266,259	580		Kidney stones^U,+^
**19p13.3**	19	6,873,527	6,881,286	8		Systemic lupus erythematosus^U,+,M^
**19p13.2**	19	11,210,904	11,218,188	7		Ischemic heart disease^−^
**20p12.1**	20	14,523,969	14,652,973	129		Gout^U^
**22q11.21**	22	19,024,651	21,463,545	2,439	Y	Aneurysm^−^, **headaches**^+,M^, **ischemic heart disease**^U,+,M^
**22q11.21-22q11.22**	22	21,797,101	22,661,627	865	*	Disorders of mineral metabolism^−,M^
**22q11.23**	22	23,627,256	23,658,006	31	*	Disorders of bilirubin metabolism^−,M^
**22q12.1**	22	25,929,538	25,994,013	64		Glaucoma^U, −^
**Xp22.33**	X	1,746,850	2,046,202	299		Sleep apnea^M^
**Xp22.33**	X	2,128,228	2,361,712	233		Disease burden^U,+,M^
**Xp22.33**	X	2,814,160	2,945,477	131		Celiac disease^U^
**Xp22.11**	X	22,946,631	23,087,940	141		Ovarian cancer^U, −,M^
**Xq28**	X	152,703,776	152,887,811	184		Ovarian cancer^U,+,M^

### Validation of identified CNV-GWAS signals

Across the 45 disease-associated CNVRs, CNV frequencies were low, ranging between 0.01% (our frequency cutoff) and 0.36%, with 87% (39/45) of CNVRs having a frequency ≤ 0.1% (Fig. 2A). Consequently, associations rely on a low number of diseased CNV carriers and require validation (Fig. 1A; bottom; Fig. 2B; Additional file 3: Table S4). We used three statistical approaches to assess the robustness of CNV-disease associations: (i) Fisher test, (ii) residual regression, and (iii) time-to-event analysis through CoxPH modeling. We replicated 28/70 (40%), 23/70 (33%), and 70/70 (100%) of the associations with the respective methods at the arbitrary validation threshold of *p* ≤ 10^−4^. This allowed to stratify associations in confidence tiers, with 17 signals replicating with all methods (tier 1), 20 with two (tier 2), and 36 only through time-to-event analysis (tier 3). Importantly, time-to-event analysis showed that CNVs always contributed to an earlier age of disease onset (Additional file 3: Table S4), in line with the paradigm that diseases with a strong genetic etiology have earlier onset [58]. Finally, when accounting for the number of assessed traits by using a stringent experiment-wide threshold for significance (*p* ≤ 1.2 × 10^−7^), 32 out of 73 (44%) CNV-GWAS signals remained significantly associated. These signals were enriched for tier 1 and 2 associations (*p*_Fisher_ = 0.05).

In parallel, we gathered literature evidence linking genetic variation at CNVRs with relevant phenotypes (Additional file 3: Table S4). Forty-eight signals (48/73 = 64%) mapped to a CNVR harboring a least one OMIM morbid gene and in 15 cases, the gene was linked to a Mendelian disorder sharing phenotypic features with the associated common disease. For instance, association between 4q35 CNVs and corneal conditions (chr4:186,687,554–187,182,384; OR_U-shape_ = 18.2; 95%-CI [5.2; 63.1]; *p* = 5.0 × 10^−6^) mapped to *CYP4V2* [MIM: 608614], a gene associated with autosomal recessive Bietti crystalline corneoretinal dystrophy [MIM: 210370], a disorder that impairs vision and progresses to blindness by age 50–60 years [59]. We next assessed whether SNPs overlapping disease-associated CNVRs were reported to associate with the implicated disease or a biomarker thereof in the GWAS Catalog. This was the case for 28 (28/66 = 42%) autosomal signals, a similar proportion (38%) than for continuous trait CNV-GWASs [27]. For instance, distal 22q11.2 CNVs increased risk for disorders of mineral metabolism (chr22:21,797,101–22,661,627; OR_mirror_ = 0.02; 95%-CI [0.006; 0.083]; *p* = 9.9 × 10^−9^) and overlapped heel bone mineral density SNP-GWASs signals, while 3q29 CNVs increased Alzheimer’s disease risk (chr3:196,953,177–197,331,898; OR_U-shape_ = 11.8; 95%-CI [4.0; 34.7]; *p* = 6.6 × 10^−6^) and overlapped with SNP-GWAS signal for PHF-tau levels, and suggestive signals (*p* < 5 × 10^−6^) for frontotemporal dementia and cognitive decline in Alzheimer’s disease. Finally, 37 signals (37/73 = 51%) mapped to nine CNVRs previously found to be associated with complex traits [27], among which eight correspond to known GDs.

**Fig. 3:**
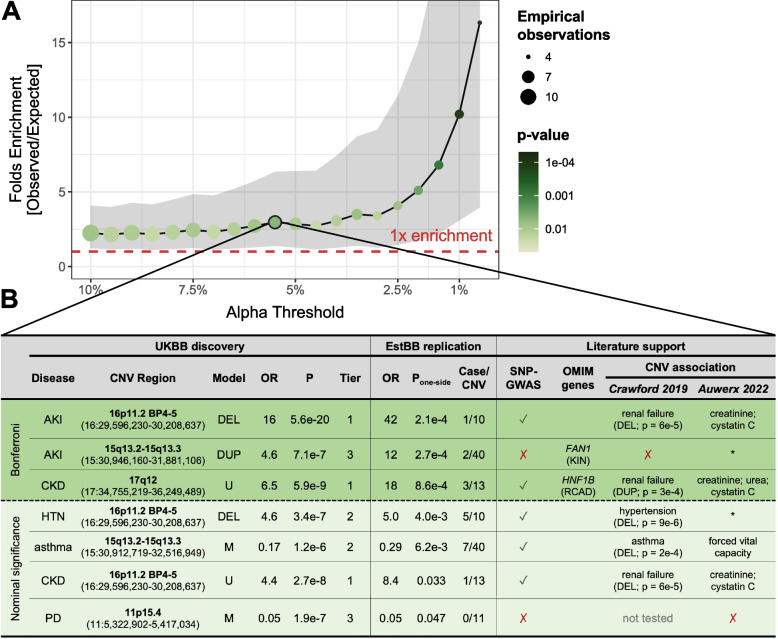
Replication of CNV-disease associations in the Estonian Biobank. **A** Enrichment for signal replication (*y*-axis; 95% confidence interval as gray ribbon) at different levels of significance (alpha; *x*-axis) in the Estonian Biobank (EstBB). Color and size indicate the *p*-value of the enrichment (one-sided binomial test) and the number of observed associations, respectively. Dashed red line indicates one-fold enrichment, i.e., the number of observed associations matches the number of expected ones. **B** Associations replicated at nominal significance in the EstBB, color-stratified according to whether they meet the replication (*p* ≤ 1.0 × 10^−3^; green) or nominal (*p* ≤ 0.05; light green) significance threshold. Disease (CKD = chronic kidney disease; AKI = acute kidney injury; HTN = hypertension; PD = Parkinson’s disease), cytogenic band and coordinates, best model (M = mirror; U = U-shape; DUP = duplication-only; DEL = deletion-only), odds ratio (OR), *p*-value (P), and statistical confidence tier are given for the UK Biobank (UKBB) discovery analysis. OR, one-sided *p*-values, and number of cases among CNV carriers are provided for the EstBB replication. Overlap with SNP-GWAS signals for a related trait (✓ = yes; ✗ = no) or a relevant OMIM gene (RCAD = renal cyst and diabetes; KIN = karyomegalic interstitial nephritis) is indicated. Previous association with diseases [24] (duplication (DUP) or deletion (DEL) was associated with indicated disease; no association (✗); some CNVRs were not tested) and continuous traits [27] (disease-relevant biomarkers are specified; other traits (*); no association (✗)) are listed

We also set out to replicate association signals in 90,211 unrelated EstBB individuals [49], using a similar case definition as in the UKBB analysis (Additional file 1: Note S5; Additional file 2: Figure S1). A total of 49 out of 73 associations could be evaluated, among which three were strictly replicated (*p* ≤ 0.05/49 = 1.0 × 10^−3^) and four additional ones reached nominal significance (*p* ≤ 0.05) (Additional file 3: Table S4). Compared to what would be expected by chance, this corresponds to a 2.9-fold (*p*_binomial_ = 0.011) and 16.3-fold (*p*_binomial_ = 1.1 × 10^−4^) enrichment for replication at *p* ≤ 0.05 and *p* ≤ 5 × 10^−3^, respectively (Fig. 3A). We have previously shown that the smaller sample size of the EstBB strongly limits replication power [27]. Hence, despite only 7 out of 49 (14%) associations being nominally replicated, the strong enrichment for significant results support validity of the primary UKBB association signals. Replicated associations harbor SNP-GWAS signals for related phenotypes (5/7), relevant morbid OMIM genes (2/7), or map to CNVRs previously associated with similar diseases (5/6) or biomarkers (4/7) (Fig. 3B). Among them is the association between 15q13 duplications and increased risk for acute kidney injury (AKI; chr15:30,946,160–31,881,106 | UKBB: OR_dup_ = 4.6; 95%-CI [2.5; 8.4]; *p* = 7.1 × 10^−7^ | EstBB: *p* = 2.7 × 10^−4^). Homozygous LoF mutations in *FAN1* [MIM: 613534], one of the five genes mapping to this CNVR, have been linked to karyomegalic interstitial nephritis [MIM: 614817] [60], opening the possibility that both increased and decreased dosage of this region have negative consequences on renal health. Importantly, integrating evidence provided by statistical, literature-based, or independent replication helps prioritize the most promising associations for follow-up studies and pinpoint plausible candidate genes.

### CNV-disease associations driven by BMI

Large recurrent CNVs have been linked to altered body weight [20, 23, 26, 27], which itself represents a risk factor for a broad range of common diseases. We identified 25 CNV-disease associations for which both disease risk and CNV status associated with BMI, indicating that the latter might confound these associations. While including BMI as an additional covariate did not result in significantly different CNV effects, 12 out of 25 associations did not meet the strict GW significance threshold anymore (Table 1; Additional file 2: Figure S2; Additional file 3: Table S5), so that 16% of the 73 associations uncovered by our CNV-GWAS are likely driven by the CNV’s propensity for increasing adiposity in its carriers. In line with expectations, associations showing the strongest confounding include cardiometabolic diseases such as lipidemia, or sleep apnea, while pulmonary, renal, and psychiatric diseases, along with the disease burden were less affected. Importantly, only one CNVR lost all its associations upon BMI adjustment, i.e., the *SH2B1*-overlapping distal 16p11.2 BP2-3 deletion, which is known to cause severe, early-onset obesity [47, 61].

### Global characterization of disease-associated CNV regions

We sought to identify global characteristics that distinguish disease-associated CNVRs (Additional file 3: Table S6). Number of protein-coding genes embedded in the 45 disease-associated CNVRs ranged from 0 to over 30 and generally correlated with the number of encompassed probes (ρ_Pearson_ = 0.50; *p* = 4.2 × 10^−4^; Additional file 2: Figure S3A). Exceptions include single-gene CNVRs overlapping well-known pathogenic genes captured thanks to high probe coverage, such as *BRCA1* (Additional file 1: Note S7) or *LDLR* (Additional file 1: Note S8). Seven CNVRs (16%) associated with multiple diseases, all of which mapped to known GD regions. One CNVR that stood out is the 600 kb 16p11.2 BP4-5 region (Fig. 2B; Table 1). Originally identified as a major risk factor for autism, schizophrenia, developmental delay and intellectual disability, macro-/microcephaly, epilepsy, and obesity/underweight [62–68], we previously found the region to associate with 26 continuous complex traits [27]. Here, we show that 16p11.2 BP4-5 deletions increase the risk of 12 diseases across multiple organ systems as well as the disease burden (+ 3 diseases/deletion; *p* = 1.2 × 10^−26^), five of which, alongside the disease burden, remain significant upon adjustment for BMI (Table 1; Additional file 3: Table S5). On the other hand, the region’s duplication drove increased risk for psychiatric conditions (i.e., bipolar disorder, schizophrenia, and depression), in line with previous findings [67].

Next, we assessed whether disease genes were under stronger evolutionary constraint than genes affected by CNVs at the same frequency but not associated with any disease (i.e., “background genes”). Compared to background genes, the 231 disease genes had more constrained pLI (*p*_Wilcoxon_ = 1.3 × 10^−4^; Additional file 2: Figure S3B) and LOEUF (*p*_Wilcoxon_ = 1.9 × 10^−7^; Additional file 2: Figure S3C) scores, suggesting stronger intolerance to LoF mutations. Splitting CNVRs depending on whether they have at least one association through either the duplication-only or deletion-only model, we evaluated whether embedded disease genes were more susceptible to haploinsufficiency (Additional file 2: Figure S3D) or triplosensitivity (Additional file 2: Figure S3E). No significant difference in pHaplo scores were observed but genes overlapping regions whose duplication (*p*_Wilcoxon_ = 9.0 × 10^−19^) and deletion (*p*_Wilcoxon_ = 1.0 × 10^−23^) have been linked to diseases were more likely to be triplosensitive than background genes. Similar trends were observed considering genes overlapping CNVRs involved uniquely through the duplication-only and deletion-only models and not the other CNV type-specific model (Additional file 2: Figure S3F-G). Overall, our results indicate that a CNVR’s pathogenicity is influenced both by the number and characteristics of affected genes, even though our study did not explore whether part of the observed phenotypic consequences is driven by disruption of regulatory regions [4].

### CNV-biomarker- associations tag pathophysiological processes

**Fig. 4:**
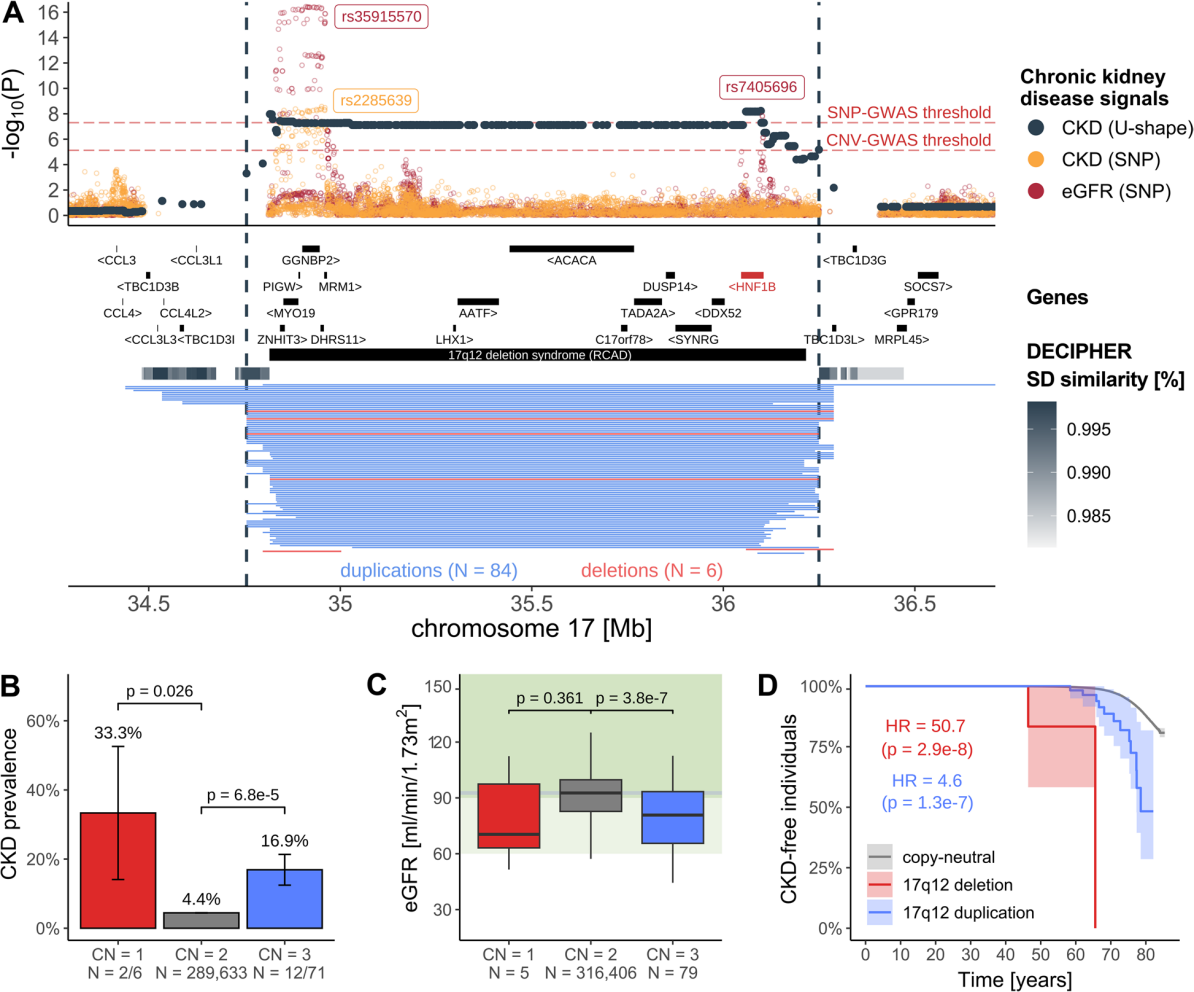
Increased and decreased dosage of 17q12 impairs kidney function. **A** 17q12 association landscape. Top: Negative logarithm of association *p*-values of CNVs (dark gray; CNV region (CNVR) delimited by vertical dashed lines) and single-nucleotide polymorphisms (SNPs) with chronic kidney disease (CKD; orange) [71] and SNPs with estimated glomerular filtration rate (eGFR; red) [72]. Lead SNPs are labeled. Red horizontal dashed lines represent the genome-wide threshold for significance for CNV-GWAS (*p* ≤ 7.5 × 10^−6^) and SNP-GWAS (*p* ≤ 5 × 10^−8^). Middle: Genomic coordinates of genes and DECIPHER GD, with *HNF1B*, the putative causal gene in red. Segmental duplications are represented as a gray gradient proportional to the degree of similarity. Bottom: Genomic coordinates of duplications (blue) and deletions (red) of UK Biobank participants overlapping the region. **B** CKD prevalence (± standard error) according to 17q12 copy-number (CN). *P*-values compare deletion (CN = 1) and duplication (CN = 3) carriers to copy-neutral (CN = 2) individuals (two-sided Fisher test). Number of cases and samples sizes are indicated (N = cases/sample size). **C** eGFR levels according to 17q12 CN, shown as boxplots; outliers are not shown. *P*-value comparisons as in **B** (two-sided *t*-test). Gray horizontal line represents median eGFR in non-carriers. Light and darker green background represent mildly decreased (60–90 ml/min/1.73m^2^) and normal (≥ 90 ml/min/1.73 m.^2^) kidney function, respectively. **D** Kaplan–Meier curve depicting the percentage, with 95% confidence interval, of individuals free of CKD over time among copy-neutral and 17q12 deletion and duplication carriers. Hazard ratio (HR) and *p*-value for deletion and duplication are given (CoxPH model)

Integration of biomarker and disease CNV-GWAS signals can identify high-confidence, clinically relevant associations. Heterozygous LoF of *HNF1B* [MIM: 189907] and 17q12 deletions cause renal cyst and diabetes (RCAD) [MIM: 137920], a severe disorder characterized by renal abnormalities and maturity-onset diabetes of the young [69, 70]. While we previously showed that renal biomarkers were increased in duplication carriers [27], here, we demonstrate that both 17q12 deletions and duplications increase CKD risk (chr17:34,755,219–36,249,489; OR_U-shape_ = 6.5; 95%-CI [3.4; 12.1]; *p* = 5.9 × 10^−9^; Fig. 4A), with a prevalence of 33.3% (*p*_*t*-test_ = 0.026) and 16.9% (*p*_*t*-test_ = 6.8 × 10^−5^) among deletion and duplication carriers, respectively, versus 4.4% in copy-neutral individuals (Fig. 4B). Results replicated in the EstBB (*p* = 8.6 × 10^−4^; Fig. 3B) and are supported by 20% of CNV carriers showing signs of kidney disease based on eGFR (< 60 ml/min/1.73m^2^), compared to 2.2% in copy-neutral individuals (Fig. 4C). Importantly, both 17q12 deletion and duplication lower age of CKD onset (HR ≥ 4.6; *p* ≤ 1.3 × 10^−7^; Fig. 4D), providing strong evidence of the deleterious consequences on kidney health of altered dosage of 17q12. These results align with two recent clinical studies that found that 17q12 deletions were observed in ~ 2% of individuals with congenital kidney anomalies [42] and that the 17q12 CNV was the most common GD etiology within a cohort of 6,679 CKD cases, in which nine deletion and seven duplication carriers were identified [43]. In another similar example, the blood pressure-increasing 16p12.2 deletion (chr16:21,946,523–22,440,319) [23, 27] increased risk for hypertension (OR_del_ = 2.7; 95%-CI [1.9; 3.8]; *p* = 1.3 × 10^−8^) and cardiac conduction disorders (OR_del_ = 3.3; 95%-CI [2.2; 4.9]; *p* = 1.1 × 10^−8^), suggesting a role in cardiovascular health (Additional file 1: Note S9) and highlighting the relevance of CNV-biomarker associations.

### Dissecting complex pleiotropic CNV regions

**Fig. 5:**
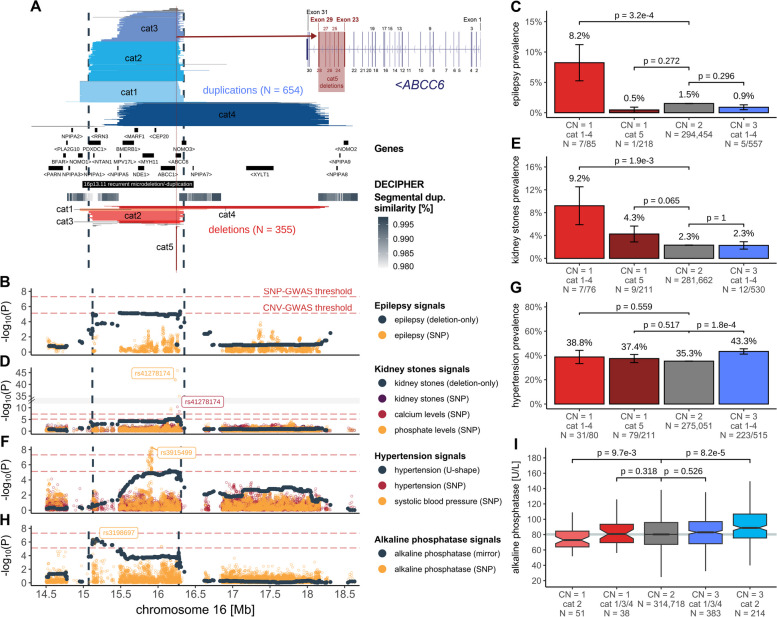
Dissection of complex pleiotropic patterns of recurrent CNVs at 16p13.11. **A** 16p13.11 genetic landscape. Coordinates of UK Biobank duplications (shades of blue; top) and deletions (shades of red; bottom) overlapping the maximal CNV region (CNVR delimited by vertical dashed lines) associated with epilepsy, kidney stones, hypertension, and alkaline phosphatase (ALP). CNVs are divided and colored according to five categories (cat1-5) to reflect recurrent breakpoints, with atypical CNVs in gray (Additional file 1: Note S6). Breakpoints reflect segmental duplications, represented with a gray gradient proportional to the degree of similarity. Middle: genomic coordinates of genes and DECIPHER GD. Inset: Overlap between *ABCC6*’s exonic structure and cat5 deletions. **B**, **D**, **F**, **H** Negative logarithm of association *p*-values of CNVs (dark gray; model in parenthesis; CNVR delimited by vertical dashed lines) with **B** epilepsy, **D** kidney stones, **F** hypertension, and **H** ALP and SNPs with **B** epilepsy [73], **D** kidney stones [74], calcium levels, and phosphate levels (*y*-axis; break: //); **F** hypertension and systolic blood pressure [75], and **H** ALP. Lead SNPs are labeled. Red horizontal dashed lines represent genome-wide thresholds for significance for CNV-GWAS (*p* ≤ 7.5 × 10^−6^) and SNP-GWAS (*p* ≤ 5 × 10^−8^). **C**, **E**, **G** Prevalence (± standard error) of **C** epilepsy, **E** kidney stones, and **G** hypertension according to 16p13.11 copy-number (CN) and CNV categories from **A**. *P*-values compare carriers of specific deletions (CN = 1) and duplications (CN = 3) to copy-neutral (CN = 2) individuals (two-sided Fisher test). Number of cases and samples sizes are indicated (N = cases/sample size). **I** ALP levels according to 16p13.11 CN and CNV category, shown as boxplots; outliers are not shown. *P*-values compare carriers of specific deletions (CN = 1) and duplications (CN = 3) to copy-neutral (CN = 2) individuals (two-sided *t*-test). Gray horizontal line represents median ALP value in non-carriers

While some CNV signals converge onto the same underlying physiological processes, others tie apparently unrelated traits to the same genetic region, suggesting genuine pleiotropy. 16p13.11 harbors multiple, partially overlapping recurrent groups of CNVs that allow fine-mapping of signals to different subregions of the CNVR (Fig. 5). Through different association models, the CNVR was linked to uncorrelated traits including epilepsy, kidney stones, hypertension, alkaline phosphatase (ALP), forced vital capacity, and age at menopause and menarche. We previously proposed *MARF1* as a candidate gene for the female reproductive phenotypes [27] and will focus here on the remaining traits.

The 654 duplications and 355 deletions overlapping the maximal CNVR (chr16:15,070,916–16,353,166) were grouped into 5 categories (cat1-5) based on their breakpoints (Fig. 5A). Matching previous findings [44], risk for epilepsy was increased in deletion carriers (chr16:15,122,801–16,353,166; OR_del_ = 6.2; 95%-CI [2.8; 13.4]; *p* = 4.4 × 10^−6^; Fig. 5B), with a prevalence of 8.2% among cat1-4 deletion carriers compared to less than 1.5% among copy-neutral and duplication carriers (Fig. 5C). Previously associated with epilepsy in clinical cohorts [7, 76, 77], the region harbors *NDE1* [MIM: 609449], a gene associated with autosomal recessive lissencephaly [MIM: 614019] and microhydranencephaly [MIM: 605013] and whose mutation has been linked to epilepsy [78, 79]. Deletions also increased risk for kidney stones (chr16:15,120,501–16,353,166; OR_del_ = 5.9; 95%-CI [2.9; 11.9]; *p* = 7.3 × 10^−7^), with the CNV-GWAS signal peaking close to a missense variant (rs41278174 G > A; Frequency_A_: 2.6%) in exon 23 of *ABCC6* [MIM: 603234] associating with calcium and phosphate levels through SNP-GWASs (Fig. 5D). These signals coincide with the recurrent cat5 deletion that covers 29 probes spanning exons 23–29 of *ABCC6* (Fig. 5A). Kidney stones prevalence reaches 4.3% among cat5 deletion carriers, in-between estimates for larger cat1-4 deletion carriers (9.2%) and copy-neutral individuals (2.3%) (Fig. 5E). A wide range of variants affecting *ABCC6* have been identified and linked to the calcification disorder pseudoxanthoma elasticum through recessive [MIM: 264800]—and more rarely dominant [MIM: 177850]—inheritance [80–83], with the *Alu*-mediated cat5 deletion representing one of the most frequent variants [84, 85]. *ABCC6* is expressed in the kidney and recent estimates from clinical cohorts suggested that kidney stones are an unrecognized (i.e., not used to establish clinical diagnosis) but prevalent (11–40%) feature of pseudoxanthoma elasticum [86–88]. Our data support kidney stones as a clinical outcome of *ABCC6* disruption with partial gene deletions having lower penetrance than larger 16p13.11 deletions. Unlike epilepsy and kidney stones, both deletion (38.8%) and duplication (43.3%) carriers are at increased risk for hypertension (chr16:15,127,986–16,308,285; OR_U-shape_ = 1.5; 95%-CI [1.3; 1.8]; *p* = 5.5 × 10^−6^; Fig. 5F), compared to copy-neutral individuals (35.3%) (Fig. 5G). The CNVR overlaps a SNP-GWAS signal for systolic blood pressure mapping to *MYH11* [MIM: 160745] (Fig. 5F). Expressed in arteries, *MYH11* encodes for smooth muscle myosin heavy chains and has been linked to dominant familial thoracic aortic aneurysm [MIM: 132900], for which hypertension represents a leading risk factor. Intermediate prevalence (37.4%) of hypertension among cat5 deletions implicates *ABCC6*, suggesting that multiple genes contribute to hypertension risk at 16p13.11. Consistent with this model, *ABCC6* plays a role in vascular calcification as the causal gene for generalized arterial calcification of infancy [MIM: 614473] [89, 90], typically diagnosed by hypertension in newborns. Interestingly, the previously described mirror association with ALP (chr16:15,070,916–16,276,964; *β*_mirror_ = 6.6 U/L; p = 3.5 × 10^−7^; UKBB field #30610) peaks at the distal end of the CNVR [27], nearby a suggestive SNP-GWAS signal for ALP levels (Fig. 5H). Splitting ALP levels by CNV category revealed that this mirroring effect is driven by individuals with cat2 deletion (mean = 76.4 U/L; *p*_*t*-test_ = 9.7 × 10^−3^) and duplication (mean = 92.9 U/L; *p*_*t*-test_ = 8.2 × 10^−5^), as other CNV carriers had ALP levels indistinguishable from those of copy-neutral individuals (mean = 83.6 U/L) (Fig. 5I). Hence, we propose the distal region of the CNVR to harbor the critical region regulating ALP levels, even though no obvious candidate gene could be identified in the literature.

**Fig. 6:**
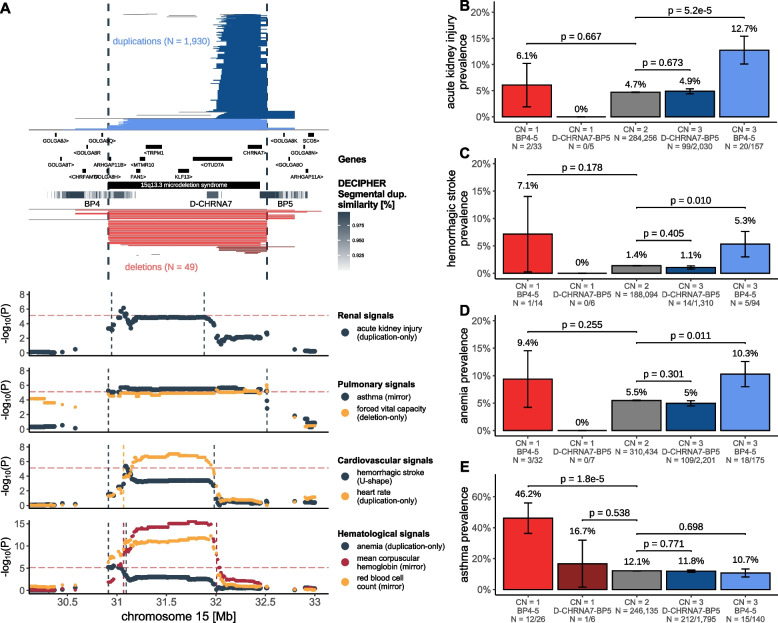
Dissection of complex pleiotropic patterns of recurrent CNVs at 15q13. **A** 15q13 genetic landscape. Top: Coordinates of duplications (shades of blue; top) and deletions (shades of red; bottom) overlapping the maximal CNV region (CNVR; delimited by vertical dashed lines) associated with acute kidney injury (AKI), asthma, forced vital capacity, hemorrhagic strokes, heart rate, anemia, mean corpuscular hemoglobin, and red blood cell count. CNVs are divided and colored according to whether they span breakpoint (BP) 4 to 5 or D-CHRNA7 to BP5, with atypical CNVs in gray (Additional file 1: Note 6). Breakpoints reflect segmental duplications, represented as a gray gradient proportional to the degree of similarity. Genomic coordinates of genes and DECIPHER GD are displayed. Bottom: Negative logarithm of association *p*-values of CNVs (best model in parenthesis) with renal, pulmonary, cardiovascular, and hematological traits. Traits-specific CNVRs are shown with vertical dashed lines. Red horizontal dashed line represents the genome-wide threshold for significance for CNV-GWAS (*p* ≤ 7.5 × 10^−6^). **B**, **C**, **D**,** E** Prevalence (± standard error) of **B** AKI, **C** hemorrhagic stroke, **D** anemia, and **E** asthma according to 15q13 copy-number (CN) and groups from **A**. *P*-values compare BP4-5 and D-CHRNA7-BP5 deletion (CN = 1) and duplication (CN = 3) carriers to copy-neutral (CN = 2) individuals (two-sided Fisher test). Number of cases and sample sizes are indicated (N = cases/sample size)

Another region exhibiting complex pleiotropic patters is 15q13. Deletions spanning BP4-5 [MIM: 612001]—and to a lesser extent duplications—have been associated with neuropsychiatric and developmental conditions [91, 92], with the nicotinic acetylcholine receptor ion channel *CHRNA7* being proposed as the driver gene based on the presence of similar phenotypes in individuals with a smaller deletion (D-CHRNA7-BP5) only affecting *CHRNA7* [93] (Fig. 6A)*.* BP4-5 duplication carriers—but not ~ 10-times more numerous D-CHRNA7-BP5 duplication carriers—showed higher prevalence of AKI (EstBB-replicated: Figs. 3B and 6B), hemorrhagic stroke (chr15:30,912,719–31,982,408; OR_U-shape_ = 7.5; 95%-CI [3.2; 17.9]; *p* = 4.3 × 10^−6^; Fig. 6C; note that this association is possibly confounded by BMI; Table 1; Additional file 3: Table S5), and anemia (chr15:30,912,719–31,094,479; OR_dup_ = 4.9; 95%-CI [2.5; 9.7]; *p* = 3.2 × 10^−6^; Fig. 6D), reminiscent of associations with pulse rate, mean corpuscular hemoglobin, and red blood cell count [23, 27]. Replicating an association with asthma [24] (chr15:30,912,719–32,516,949; OR_mirror_ = 0.17; 95%-CI [0.08; 0.35]; *p* = 1.2 × 10^−6^) which parallels the previously reported decreased forced vital capacity [27] and peak expiratory flow [23], this was the only association at the locus driven by deletions, with prevalence being increased in only BP4-5 (46.2%; *p*_*t*-test_ = 1.8 × 10^−5^) but not D-CHRNA7-BP5 deletion carriers (16.7%; *p*_*t*-test_ = 0.538), compared to copy-neutral individuals (12.1%) (Fig. 6E). Hence, the non-neurological disorders we associate with 15q13 CNVs appear to specifically involve dosage of the genes within BP4-D-CHRNA7 and not *CHRNA7*.

### CNV burden at known genomic disorder CNVRs increases overall disease risk

**Fig. 7:**
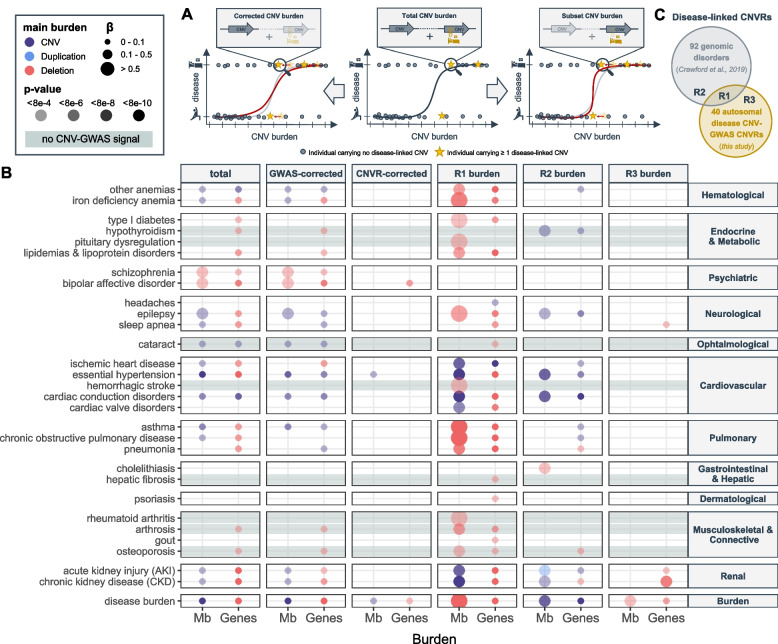
CNV burden at known genomic disorder CNVRs increases overall disease risk. **A** Burden calculation. Middle: Total CNV (duplication + deletion), duplication, or deletion burdens are calculated by summing up the length (in number of affected Mb or genes) of all CNVs, duplications, or deletions in an individual, respectively. Burden values are used as a predictor for disease risk. Left: Corrected burdens are calculated by summing up the length of all CNVs, duplications, or deletions that do not overlap with regions listed in a given genomic partition. Right: Subset burdens are calculated by summing up the length of all CNVs, duplications, or deletions that overlap with regions listed in a given genomic partition. Both corrected and subset burden values are used to re-estimate contribution of the CNV burden to disease risk (red curve). **B** Contribution of the total burden, CNV-GWAS signal- and CNVR-corrected burdens, and the R1, R2, and R3 subset burdens measured in number of affected Mb (*x*-axis; left) or genes (*x*-axis; right) to disease risk (*y*-axis). Only the effect of the most significantly associated of the CNV (purple), duplication (blue), or deletion (red) burdens, providing *p* ≤ 0.05/61 = 8.2 × 10^−4^, is shown. Color indicates whether the CNV, duplication, or deletion burden was most significantly associated, with size and transparency being proportional to the effect size (beta) and *p*-value, respectively. Gray horizontal bands mark traits with no CNV-GWAS signal. **C** Schematic representation of the R1, R2, and R3 partitions used to define the subset burdens in **B**

By aggregating CNVs into a burden, we capture the effect of ultra-rare CNVs (frequency ≤ 0.01%), as well as those whose effect is not strong enough to reach GW significance under current settings, increasing our power to detect the global pathogenic impact of CNVs on human health. Individual-level autosomal CNV (duplication + deletion), duplication, and deletion burdens were calculated as the number of Mb or genes affected by the considered type of CNV. The predictive value of these six CNV burden metrices on the same 60 diseases (and the disease burden) previously assessed through CNV-GWAS was estimated (Fig. 7A; “middle”). Disease burden strongly associated with a high CNV load (*β*_del_ =  + 0.03 disease per deleted gene; *p* = 3.7 × 10^−27^) and risk for 20 individual disorders was increased by at least one type of CNV burden (*p* ≤ 0.05/61 = 8.2 × 10^−4^; Fig. 7B; “total burden”; Additional file 3: Table S7). Overall, the deletion burden tended to yield more significant associations than the duplication burden and strongest effect sizes were observed for psychiatric disorders, such as bipolar disorder (OR_Mb_del_ = 1.4; *p* = 6.9 × 10^−4^), schizophrenia (OR _Mb_del_ = 1.4; *p* = 4.1 × 10^−5^), or epilepsy (OR_Mb_CNV_ = 1.1; *p* = 8.3 × 10^−5^), in agreement with CNVs representing important risk factors for these complex and polygenic disorders. Still, we note that the CNV burden only accounts for ~ 0.02% of the variability in disease burden, with up to 0.1% of schizophrenia and bipolar disorder cases being explained by the CNV burden (Additional file 3: Table S8).

To ensure that we do not merely capture the effect of individual CNV-disease associations previously isolated by CNV-GWAS, we corrected the CNV burdens for CNV-GWAS signals. Specifically, we excluded from the burden calculation CNVs overlapping disease-associated CNVRs in a disease- and burden-type-specific fashion. We then estimated the predictive value of these corrected burdens on disease risk (Fig. 7A; left). Overall association strength dropped but signal was lost only for type 1 diabetes and chronic obstructive pulmonary disease (Fig. 7B; “GWAS-corrected”; Additional file 3: Table S7). However, if we exclude CNVs overlapping the 40 autosomal unique disease-associated CNVRs systematically, i.e. not in a disease- and burden-type-specific fashion, the bulk of association signals disappears (Fig. 7B; “CNVR-corrected”; Additional file 3: Table S7), indicating that the genomic partition uncovered by our CNV-GWAS increases disease risk beyond the 73 CNV-disease pairs that reach genome-wide significance.

To further explore this hypothesis, we calculated subset CNV burdens (Fig. 7A; right) overlapping three different genomic partitions (Fig. 7C) composed of (i) nine disease-associated CNVRs that map to known GDs (R1), (ii) regions of known GDs that did not yield any association in our CNV-GWAS (R2), (iii) and disease-associated CNVRs uncovered by our CNV-GWAS that were not linked to a known GD (R3). Risk for 25 diseases, as well as the disease burden, were significantly increased by the R1 CNV burden subset and included associations with eight diseases that were not picked up by the total burden association (Fig. 7B; “R1 burden”; Tables S7). We observed a substantial contribution of the R2 burden subset to the risk of diseases such as epilepsy, hypertension, cardiac conduction disorders, AKI, CKD, and hypothyroidism, even though the pleiotropy of this partition was more moderate than the one of the R1 burden subset (Fig. 7B; “R2 burden”; Tables S7). Few associations were observed for the R3 CNV burden (Fig. 7B; “R3 burden”; Tables S7). Supporting these results, the CNVR (R1 + R3 partitions) and GD (R1 + R2 partitions) burden subsets strongly associate with 28 and 23 phenotypes, respectively (Additional file 2: Figure S4; Additional file 3: Table S7). A gradual loss of the number of associations was found when correcting the total CNV burden for the R3, R2, R1, GD, and CNVR partitions, with similar trends observed when requiring a more stringent overlap between CNVs and defined regions (“Methods”; Additional file 2: Figure S4; Additional file 3: Table S7). Overall, our results indicate that known GD CNVRs are the major drivers of the CNV burden’s pathogenicity and hint at their currently underestimated pleiotropy.

## Discussion

Using an adapted GWAS framework, we provide a detailed investigation of the contribution of rare CNVs to the genetic architecture of 60 common diseases and showcase how the rich phenotypic data of the UKBB can be leveraged to gain new biological insights, highlighting the role of CNVs as modulators of common disease susceptibility in the general population.

Various strategies have been used to study CNV-disease associations in the UKBB. Focusing on diseases related to the ones assessed in the current study, we replicate 10 out of the 24 detected associations (at FDR ≤ 0.1) with 54 likely pathogenic CNVs [24] and all four associations (at *p* ≤ 1 × 10^−9^) in a recent CNV-GWAS investigating 757 diseases [21]. Despite data originating from the same cohort, we often obtained *p*-values orders of magnitude smaller (e.g., 16p11.2 BP4-5 deletion and AKI: *p* = 5.6 × 10^−20^; *p* = 6.0 × 10^−5^ [24]; *p* = 3.3 × 10^−15^ [21]). The increased power of our study might be explained by accruing case count from updated hospital records, careful case–control definition and statistical handling of the binary outcomes, probe-level association analysis, and usage of different association models to mimic various dosage mechanisms. We consequently identified previously unreported CNV-disease associations whose relevance was asserted by follow-up analyses. Only one signal—17q12 CNVs increasing CKD risk—was backed by all approaches, emphasizing the importance of considering diverse lines of corroborative evidence, such as overlap with relevant SNP-GWAS signals and OMIM genes that indicate shared genetic mechanisms or both disease and disease-relevant biomarker associations mapping to the same CNVR. For instance, four (1q21.1–1q21.2, 15q13, 16p12.2, 16p11.2 BP4-5) out of six CNVRs decreasing forced vital capacity [27] were found to increase risk for pulmonary diseases, with the association between 15q13 and asthma replicating in the EstBB (*p* = 6.2 × 10^−3^) and 16p11.2 BP4-5 CNVs carriers being found to be enriched for “abnormal findings examination of lungs” in the Vanderbilt University Medical Center electronic health record database [48]. This demonstrates that biomarkers are efficient proxies underlying (CNV-driven) pathological processes, often increasing the statistical power to detect associations due to their continuous nature. While we regressed covariates out of disease status to render the outcome quantitative, more sophisticated approaches have recently been developed for SNP-based GWASs that transform binary outcomes into continuous liability scores while borrowing information from age of disease onset, sex, and familial history [94]. Future exploration is warranted to assess the benefit of this approach in the context of CNV-GWASs. By coupling a CNV-GWAS framework that accounts for challenges linked to disease CNV association studies in population cohorts to extensive validation, we generated a list of 73 CNV-disease pairs with various levels of supporting evidence that can inform follow-up studies.

Disease-associated CNVRs harbored genes under stronger evolutionary constraint than those lacking associations and their length correlated with their propensity for pleiotropy, indicating that as previously observed [9], both the number and the nature of genes affected by CNVs influence their pathogenicity. Consequently, large, multi-gene, recurrent CNVs exhibited the strongest pleiotropy. A longstanding question relates to the identification of causal genes whose altered dosage drives the phenotypic alterations observed in carriers. Models with various levels of complexity have been proposed, ranging from a single driver gene to multiple driver genes modulated by epistatic interactions with other genes in the CNVR [95]. By analyzing disease prevalence in subsets of CNV carriers, association signals could be fine-mapped to narrower regions, pinpointing candidate drivers—such as *ABCC6* for kidney stones. In other cases, our data suggests that multiple subregions of the CNVR contribute to increased risk for a given disease, as observed for 22q11.2 and ischemic heart disease (Additional file 1: Note S10) or 16p13.11 and hypertension. Interestingly, the putative driver for phenotypes originally associated with a CNVR might not be driving our newly identified associations, as shown for the 15q13 CNVR, whose non-neurological phenotypes do not appear to be linked to altered dosage of *CHRNA7*. Beyond characterizing the pleiotropic pathological consequences of recurrent CNVRs, we demonstrate that dissection of CNV-GWAS signals can fine-map associations and provide mechanistic insights into their phenotypic expression.

We show that rare CNVs, such as the ones assessed in our study, only contribute marginally (0.02%) to the global disease burden in the general population. Still, from a personalized medicine perspective, these variants are highly relevant. Indeed, all detected CNV-disease associations pointed at CNVs increasing disease risk and leading to an earlier age of onset. Incorporating age of onset information has been shown to improve power to detect associations [94], and more importantly, represents proof of clinical relevance. Many signals mapped to regions whose genetic perturbation has been reported to be pathogenic in an autosomal dominant fashion. These include associations between well-described, clinically relevant gene-disease pairs—such as *BRCA1* and *LDLR* deletions increasing the risk for early-onset ovarian cancer (Additional file 1: Note S7) and ischemic heart disease (Additional file 1: Note S8), respectively—but for which the role of CNVs in a large population cohort had not been previously investigated. CNVs in these genes have high penetrance but are extremely rare in the UKBB. Follow-up analyses based on the medical records, family history, medication use, and biomarkers could recapitulate additional clinical associations and establish that these deletions were most likely inherited. By recovering known gene-disease pairs typically studied in clinical cohorts, we showcase how the rich phenotypic data from biobanks can generate insights into the mechanisms, epidemiology, and comorbidities of these diseases, implicating CNVs as important genetic risk factors. We also highlight several examples where deviations by one copy-number are linked to common diseases which share clinical features with rare Mendelian conditions caused by homozygous perturbations of the same genetic region. For instance, risk for kidney stones is gradually increased in carriers of partial versus full *ABBC6* deletions. Another intriguing example is the association between a relatively common CNV (frequency = 0.22%) affecting exon 2 and intron 2–3 of *PRKN* [MIM: 602544]—a gene causing juvenile autosomal recessive Parkinson’s disease [MIM: 600116]—and sleep disorders such as insomnia and hypersomnia. As sleep disturbances are among the earliest symptoms of Parkinson’s disease [96], follow-up studies should determine whether these individuals are more prone to develop Parkinson’s disease. Overall, this argues against a dichotomic view on dominant versus recessive modes of inheritance and analogously to allelic series [32–35], suggests that Mendelian and common diseases represent different ends of the phenotypic spectrum caused by genetic variation at a given locus. We further show that nine CNVRs previously linked to pediatric GDs also increased risk for a broad spectrum of adult-onset common diseases. These associations were probably overlooked as the medical consequences in adulthood of these etiologies are often poorly characterized owing to ascertainment bias and difficulty to gather large cohorts. Importantly, 12 out of 24 associations mapping to a GD linked to altered BMI remained significant when accounting for the latter. This indicates that while part of the increased disease risk among individuals with GDs represents a mere comorbidity of obesity, other BMI-independent mechanisms further contribute to the high disease burden observed in these individuals. In the future, it will be important to assess the role of other possible confounders, such as clinical biomarkers or socioeconomic status, as such knowledge can guide preventive strategies and improve understanding of disease mechanisms. While awaiting validation in clinical cohorts of CNV carriers, we hope that these findings will improve clinical characterization of GDs, thereby facilitating diagnosis and allowing physicians to anticipate later-onset comorbidities. For instance, we found carriers of 16p13.11 deletions affecting *ABCC6*, the causal gene for pseudoxanthoma elasticum, to be at increased risk for kidney stones, paralleling reports from clinical cohorts showing that kidney stones represent an unrecognized feature of the disease [86–88]. Awareness of this disease feature can mitigate kidney stone risk through adapted diet and sufficient water intake. Together, our results advocate for a complex model of variable CNV expressivity and penetrance that can result in a broad range of phenotypes along the rare-to-common disease spectrum and represent fertile ground for in-depth, phenome-wide studies aiming at better characterizing specific CNV regions [45, 47].

Corroborating the deleterious impact of rare CNVs on an individual’s health parameters, socioeconomic status, and lifespan [21, 22, 26, 27, 33, 97–100], we here speculate that the CNV burden acts on the latter by increasing risk for a broad range of common diseases beyond their known role in neuropsychiatric disorders [5–8]. While both duplications and deletions contributed to increased disease risk, the deletion burden’s impact was much stronger—especially for metabolic, psychiatric, pulmonary, and musculoskeletal diseases—in line with the commonly accepted view that deletions tend to be more deleterious. While only a marginal fraction of the CNV burden’s contribution to disease risk was captured by CNV-GWAS signals, burden associations were mainly driven by known GDs. Only psychiatric disorders and the disease burden retained a significant association with the CNV burden when accounting for GDs, highlighting the polygenic CNV architecture of these traits. Illustrating the added value of the burden analysis, nine diseases showed a burden association despite lacking any CNV-GWAS signal. In some cases, such as for hypothyroidism, the burden signal originated from GDs that did not yield any significant CNV-GWAS associations, possibly because the involved regions did not pass the ≥ 0.01% CNV frequency filter. In other cases, such as for osteoporosis, the signal appeared to emanate from the CNVRs pick-up by the CNV-GWAS, indicating that we were likely underpowered to detect associations with any specific region. Overall, a total of 49 (82%) of the assessed diseases associated with CNVs either through CNV-GWAS or burden analysis, emphasizing the important role of this mutational class. While our burden analysis revealed that these associations mainly stem from known GDs, it also highlights that the latter are even more pleiotropic than what appears from our CNV-GWAS, implying that increased power will broaden the spectrum of common diseases associated with rare GDs.

A major limitation of our study is the reliance on microarray CNV calls, which allows us to assess only a fraction of the CNV landscape, i.e., mostly large CNVs or in regions with high probe coverage. Furthermore, as different population cohorts are genotyped with different arrays, partial probe overlap hinders replication power in external biobanks, as well as the ability to meta-analyze summary statistics. We speculate that small and/or multiallelic CNVs that can only be uncovered by sequencing will have a genetic architecture closer to the one of SNPs and indels, with higher frequencies and more subtle effect sizes. These effects, however, are more likely tagged by common variants, limiting novel discoveries. Furthermore, by detecting more events, sequencing-based studies require adapted and more stringent significance thresholds. Still, having improved breakpoint resolution, such CNV calls are also likely to enhance fine-mapping strategies. Microarray CNV calls also exhibit high false positive rates [51]. By using stringent CNV selection criteria, we decrease the latter at the cost of decreasing power to detect true associations. This aspect is particularly relevant given that the type of CNVs we assess are rare and that the UKBB is not enriched for disease cases [36], resulting in low-powered GWASs. While we adopt strategies to counter the lack of power, our results are likely subject to Winner’s curse, only capturing a fraction of the strongest, possibly overestimated effects. This phenomenon might be compensated by UKBB CNV carriers being at the milder end of the clinical spectrum, leading to effect underestimation. An interesting question will be to compare effect sizes from population-based studies to those emerging from clinical cohorts. In the future, longitudinal follow-up of UKBB participants will increase the number of cases—especially for late-onset diseases such as Alzheimer’s or Parkinson’s diseases—allowing better powered CNV-GWASs. Larger and more diverse biobanks linking genotype to phenotype data [101–103] should both validate reported associations and identify new ones.

## Conclusions

Our study provides in-depth analysis of the role of rare CNVs in modulating susceptibility to 60 common diseases in the general population, broadening our view on how this class of mutations impacts human health. Besides describing clinically relevant and actionable associations, we illustrate how complex pleiotropic patterns can be dissected to gain new insights into the pathological mechanisms of large recurrent CNVs, providing a framework that can be applied to an even larger spectrum of diseases.

### Supplementary Information

**Additional file 1: Supplemental Notes.** **Note S1.** Microarray-based CNV calling. **Note S2.** Sample filtering criteria. **Note S3.** Probe and covariate selection for main GWAS analysis. **Note S4.** Post-CNV-GWAS summary statistics processing. **Note S5.** Estonian Biobank replication. **Note S6.** Subgrouping of CNV carriers. **Note S7.** *BRCA1* deletion association with ovarian and other female cancers. **Note S8.** *LDLR* deletion association with ischemic heart disease. **Note S9.** 16p12.2 deletion associations. **Note S10.** 22q11.2 CNV associations.

**Additional file 2: Supplemental Figures.** **Figure S1.** Case-control distribution in the UK and Estonian Biobanks. **Figure S2.** BMI adjustment for possibly confounded CNV-disease associations. **Figure S3.** Constraint analysis of disease associated CNV regions. **Figure S4.** Total, corrected, and subset burden analysis.

**Additional file 3: Supplemental Tables.** **Table S1.** Disease epidemiology. **Table S2.** Covariate selection and probe prefiltering. **Table S3.** Genomic inflation of genotypic Fisher test p-values. **Table S4.** Genome-wide significant CNV-GWAS associations. **Table S5.** BMI adjustment for possibly confounded CNV-disease associations. **Table S6.** CNV region characteristics. **Table S7.** Impact of the CNV burden on disease risk. **Table S8.** Disease variance explained by the CNV burden.

## Data Availability

UKBB and EstBB data are available for registered users. Other data are freely accessible: Recurrent CNV coordinates: DECIPHER (https://www.deciphergenomics.org/) [10]; Morbid genes: OMIM (https://www.omim.org/) [104]; SNP-GWAS summary statistics for CNVR annotation: NHGRI-EBI GWAS Catalog (https://www.ebi.ac.uk/gwas/) [105]; SNP-GWAS summary statistics for locus zoom plots: epilepsy [73], eGFR [ 72], CKD [71], kidney stones [74], else Neale Lab (http://www.nealelab.is/uk-biobank) [106]; Allele frequency and gene constraint: GnomAD (https://gnomad.broadinstitute.org/) [107] and [9]; Tissue-specific gene expression: GTEx (https://gtexportal.org/home/) [108]. Generated data are available as Additional file 3 : Tables S1-S8, except for the UKBB CNV-GWAS summary statistics that are deposited on the GWAS Catalog (accession numbers: GCST90297568-GCST90297771). Code used in this study is available on GitHub (https://github.com/cauwerx/CNV_GWAS_common_diseases) [109].

## Abbreviations

ALP: Alkaline phosphatase
AKI: Acute kidney injury
BMI: Body mass index
BP: Breakpoint
CKD: Chronic kidney disease
CN: Copy-number
CNV: Copy-number variation
CNVR: CNV region
CoxPH: Cox proportional hazards
eGFR: Estimated glomerular filtration rate
EstBB: Estonian Biobank
GD: Genomic disorder
GW: Genome-wide
GWAS: Genome-wide association scan
HR: Hazard ratio
ICD-10: International Classification of Diseases, 10th Revision
LOEUF: LoF observed over expected upper bound fraction
LoF: Loss-of-function
OR: Odds ratio
PC: Principal component
pHaplo: Probability of haploinsufficiency
pLI: Probability of LoF intolerance
pTriplo: Probability of triplosensitivity
QS: Quality score
RCAD: Renal cyst and diabetes
SNP: Single-nucleotide polymorphism
UKBB: UK Biobank
