# Supplementary material for: Rare copy-number variants as modulators of common disease susceptibility
Source: Genome Med. 2024 Jan 8;16:5. doi: 10.1186/s13073-023-01265-5 (PMC10773105; doi:10.1186/s13073-023-01265-5)
Supplement: Supplementary file 1 — Additional file 1: Supplemental Notes. Note S1. Microarray-based CNV calling. Note S2. Sample filtering criteria. Note S3. Probe and covariate selection for main GWAS analysis. Note S4. Post-CNV-GWAS summary statistics processing. Note S5. Estonian Biobank replication. Note S6. Subgrouping of CNV carriers. Note S7. BRCA1 deletion association with ovarian and other female cancers. Note S8. LDLR deletion association with ischemic heart disease. Note S9. 16p12.2 deletion associations. Note S10. 22q11.2 CNV associations. [file 13073_2023_1265_MOESM1_ESM.pdf]

Additional file 1: Supplemental Notes

Rare copy-number variants as modulators of common disease susceptibility

Chiara Auwerx[1,2,3,4,*], Maarja Jõeloo[5,6], Marie C. Sadler[2,3,4], Nicolò Tesio[1], Sven Ojavee[2,3], Charlie J. Clark[1], Reedik Mägi[6], Estonian Biobank Research Team[6,§], Alexandre Reymond[1,#,*] & Zoltán Kutalik[2,3,4,#,*]

[1] Center for Integrative Genomics, University of Lausanne, 1015 Lausanne, Switzerland
[2] Department of Computational Biology, University of Lausanne, 1015 Lausanne, Switzerland
[3] Swiss Institute of Bioinformatics, 1015 Lausanne, Switzerland
[4] University Center for Primary Care and Public Health, 1005 Lausanne, Switzerland
[5] Institute of Molecular and Cell Biology, University of Tartu, 51010 Tartu, Estonia
[6] Estonian Genome Centre, Institute of Genomics, University of Tartu, 51010 Tartu, Estonia
[§] Estonian Biobank Research Team: Tõnu Esko, Andres Metspalu, Lili Milani, Reedik Mägi, Mari Nelis
[#] These authors jointly supervised this work.

*** Correspondence:**
Chiara Auwerx: chiara.auwerx@unil.ch;
Alexandre Reymond : alexandre.reymond@unil.ch;
Zoltán Kutalik: zoltan.kutalik@unil.ch.

Note S1. Microarray-based CNV calling.
Note S2. Sample filtering criteria.
Note S3. Probe and covariate selection for main GWAS analysis.
Note S4. Post-CNV-GWAS summary statistics processing.
Note S5. Estonian Biobank replication.
Note S6. Subgrouping of CNV carriers.
Note S7. *BRCA1* deletion association with ovarian and other female cancers.
Note S8. *LDLR* deletion association with ischemic heart disease.
Note S9. 16p12.2 deletion associations.
Note S10. 22q11.2 CNV associations.

Note S1: Microarray-based CNV calling

Chromosome X CNVs

Chromosome X CNVs were called using dedicated PennCNV modalities as previously described [1]. To avoid interference between the two-letter CNV encoding (Note S1 – Table 1) and the male chromosome X hemizygosity assumption of PLINK, all individuals are (falsely) labeled as female when performing genetic analyses in PLINK.

Sample CNV quality-control

Samples on genotyping plates with a mean CNV count per sample > 100 and samples with > 200 CNVs or single CNV > 10 Mb were excluded, as these might stem from artifactual CNVs consecutive of poor-quality genotyping or extreme CNV events (e.g., aneuploidies or chromothripsis) with potentially extreme phenotypic consequences that are out of the scope of this study.

CNV encoding in PLINK

CNV matrices were encoded into three PLINK binary file sets (`--make-bed` PLINK v1.9; Note S1 – Table 1) as previously described [1]. To reduce file size and facilitate parallelized computation, files are split at the chromosome level (i.e., for each PLINK encoding there are 24 files: 22 autosomes + pseudoautosomal regions + chromosome X). PLINK file sets were used to fit four association models mimicking different modes of CNV action: mirror, U-shape, duplication-only, or deletion-only (*Main CNV-GWAS model*).

Association models	Mirror		U-shape		Duplication-only		Deletion-only	
PLINK file set	$PLINK_{CNV}$		$PLINK_{CNV}$		$PLINK_{DUP}$		$PLINK_{DEL}$	
Encoding	Num.	PLINK	Num.	PLINK*	Num.	PLINK	Num.	PLINK
Deletion (QS < -0.5)	-1	AA	1	AA	NA	00	1	TT
Copy-neutral (|QS| ≤ 0.5)	0	AT	0	AT	0	AT	0	AT
Duplication (QS > 0.5)	1	TT	1	TT	1	TT	NA	00

Note S1 – Table 1. Encoding of CNVs
Encoding of high-confidence CNVs (|QS| > 0.5) into numerical probe-by-sample matrices (Num.) and three PLINK file set (PLINK), mimicking encoding of single-nucleotide variants. *The U-shape model uses the same PLINK file set as the mirror model but is assessed with the `hetonly` modifier of PLINK's `glm` function, allowing to compare the effect of deletion and duplications against copy-neutral individuals. QS = quality score.

Note S2: Sample filtering criteria

Starting from 488,377 samples, we applied several filters to obtain our final set of 331,522 individuals included for the CNV-GWAS analysis (Note S2 – Table 1).

STEP	Filter	Description	$N_{excluded}$	$N_{remaining}$
**START**				**488,377**
1	Relatedness	Samples were excluded if they were set to 0 in "`used.in.pca.calculation`" (i.e., not used for principal component analysis (PCA) calculation) in the sample QC file (v2) described in UKBB resource 531. PCA were calculated based on unrelated individuals (KING software [2] `--related --degree 3`), with missing rate on autosomes ≤ 0.02, and no mismatch between inferred and self-reported sex [3]. Focusing on unrelated individuals allows to prevent p-value deflation due to correlated residual noise.	81,158	407,219
2	Ancestry	Only sample with "*`in.white.British.ancestry.subset`*" set to 1 (i.e., self-identify as "White British" and cluster with that group based on SNP PCA analysis [3]) in the sample QC file (v2) described in UKBB resource 531 were retained. We refer to this subgroup as "white British" for the remainder of the study. This allows to obtain a sample with homogenous genetic ancestry.	69,674	337,545
3	Retracted	Samples that were redacted or retracted their participation at the time the project was initiated (August 2020) were excluded.	80	337,465
4	Genotype plate outliers	Samples that were genotyped on a genotyping plate with a mean CNV count per sample > 100 were excluded as this might indicate systematic error during the genotyping and lead to the inclusion of artifactual CNV calls.	569	336,896
5	Extreme CNV profile	Individuals with an extreme CNV profile, i.e., over 200 CNVs/sample or a single CNV larger than 10Mb were excluded. The former could either indicate poor quality genotyping, presence of a large CNV that was called as many small CNVs or extreme events such as chromothripsis. Extremely large CNV can reflect aneuploidies or other extreme chromosomal aberrations. As we expect these events to be rare, with potentially massive phenotypic consequences, we decided to exclude these individuals.	924	335,972
6	Blood cancer	Individuals with a known blood malignancy (i.e., UKBB field #20001: 10047, 1048, 1050, 1051, 1052, 1053, 1055, 1056, 1056; #41270: ICD-10 codes mapping to PheCode exclusion range "*cancer of lymphatic and hematopoietic tissue*" [4]) were excluded as these individuals are likely to harbor somatic CNVs, which are not within the scope of this study.	4,450	331,522
**END**				**331,522**

Note S2 – Table 1. Summary of sample filtering procedure
List of filters applied to generate the set of 331,522 individuals used for CNV-GWAS analysis. "STEP" indicates the order in which filters were applied, with description of the exact criteria and rationale in "Description". For each step, the number of excluded individuals ($N_{excluded}$), along with the number of remaining individuals after applying the filter ($N_{remaining}$) is indicated.

Note S3: Probe and covariate selection for main GWAS analysis

Relevant covariates and probes were pre-selected to fit tailored main CNV genome-wide association scan (GWAS) models and reduce computation time.

Covariate selection

For each disease, a logistic regression was fitted to explain disease probability as a function of age (#21003), sex (self-reported + genetically confirmed), genotyping array, and the 40 first principal components (PCs) from the single nucleotide polymorphism (SNP) genotyping data. Nominally significantly associated covariates ($p \leq 0.05$) were retained for the main GWAS. Number of retained covariates ranged between two (sarcoidosis and multiple sclerosis) and 24 (hypertension, arthrosis, and disease burden) (Note S3 – Figure 1A) and correlated with case number of the disease, aligned with the expected gain in power for more frequent diseases (Note S3 – Figure 1B). Covariates used for the main CNV-GWAS are listed in Additional file 2: Table S2.

Probe-level CNV frequency estimation and filtering

Probe-level CNV frequency was calculated as described in [1]. Briefly, for the 740'434 probes stored in $\text{PLINK}_{\text{CNV}}$ (Note S1), we counted the number of times a genotyped probe was found in a deleted (N_{del}), copy-neutral ($N_{neutral}$), and duplicated (N_{dup}) state among a subset of 331,522 selected individuals (`--freqx` PLINK v1.9). We excluded 41'670 array-specific probes with genotype count missingness > 5%. For the remaining probes, we calculated the probe-level CNV ($\frac{N_{CNV}}{N_{CNV}+N_{neutral}}$), duplication ($\frac{N_{dup}}{N_{CNV}+N_{neutral}}$), and deletion ($\frac{N_{del}}{N_{CNV}+N_{neutral}}$) frequencies, with $N_{CNV} = N_{dup} + N_{del}$. Probes with a CNV frequency < 0.01% were excluded.

Probe pruning based on copy-number status correlation

The 70,631 probes with a CNV frequency ≥ 0.01% were pruned at $r^2 > 0.9999$ in $\text{PLINK}_{\text{CNV}}$ (`--indep-pairwise 500 250 0.9999` PLINK v2.0), based on their CNV genotype, resulting in 18,725 probes. Pruning at such a high threshold will retain only a single probe at the core of a CNV region, where due to the recurrent nature of CNVs the correlation is extremely high. However, it will retain multiple probes around the CNV breakpoints (BPs), where we expect variability due to true biological variation or uncertainty of the CNV calling algorithm.

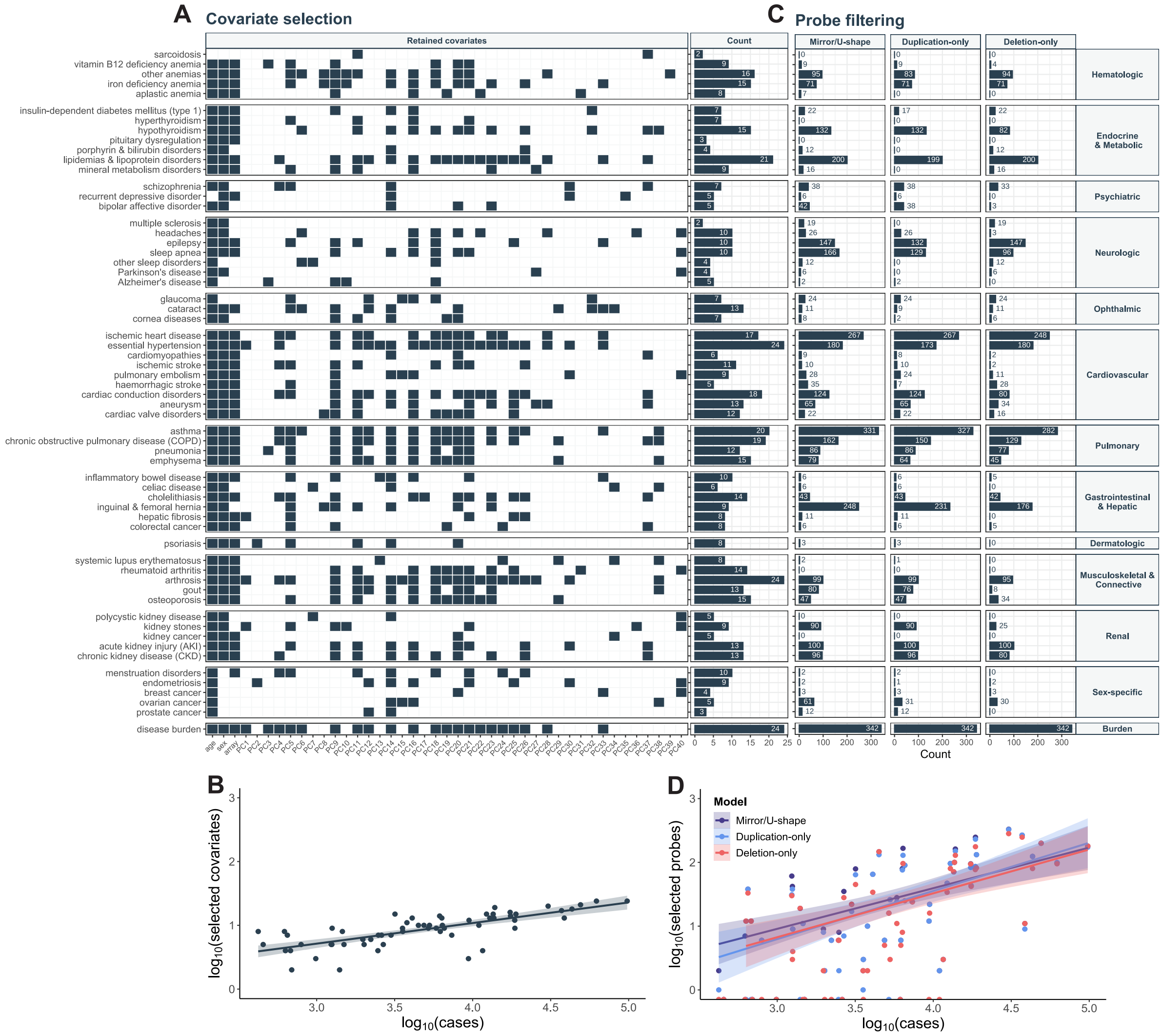

Note S3 - Figure 1. Covariate selection and probe filtering

(**A**) Left: Dark gray tiles indicate covariates (x-axis) retained for the corresponding disease and/or disease burden (y-axis) (nominal significant association). PC = principal component. Right: Number of retained covariates per disease. (**B**) Logarithm of number of selected covariates (y-axis) against the logarithm of number of cases (x-axis) for each of the 60 assessed diseases. Linear regression equations with 95% confidence intervals are displayed. (**C**) Number of probes retained after filtering (x-axis) for the mirror and U-shape (left), duplication-only (middle), and deletion-only (right) models for each of the 60 investigated diseases and the disease burden (y-axis). (**D**) Logarithm of number of selected probes (y-axis) against the logarithm of number of cases (x-axis) for each of the 60 assessed diseases, split by association model. Linear regression equations with 95% confidence intervals are displayed.

Probe selection

For each disease, 2-by-3 genotypic Fisher tests assessed dependence between disease status and probe copy-number (rows: control versus case; columns: deletion versus copy-neutral versus duplication; `--model fisher` PLINK v1.9; TEST column `GENO`). For each phenotype, QQ plots were generated by plotting the observed against the expected negative logarithm of the Fisher's test p-value. For the disease burden, p-values from linear regression were used instead. The genomic inflation factor, λ, was calculated as the median of the chi-squared test statistics derived from the Fisher's tests p-values divided by the expected median

of the chi-squared distribution. Overall, there was no sign of strong p-value inflation (Note S3 – Figure 2A). λ values above 1.1 indicate genomic inflation, which can be caused by population structure, linkage disequilibrium, or polygenicity [5] and was observed only for 6 highly polygenic traits, with a maximum value of 1.33 for the disease burden. On the other hand, 42 traits exhibited λ values below 0.9. Deflated p-values can be caused by extremely rare variants. To verify this hypothesis, λ values were calculated anew, excluding probes with the 5-80% lowest CNV frequency (in incremental steps of 5%), to determine the impact of CNV frequency on genomic factor deflation (Note S3 – Figure 2B). We observed a trend of increasing λ values when excluding low frequency probes, indicating that the deflation is indeed caused by probes with low CNV frequency. Importantly, low λ values do not increase false positive rates. λ values are available in Additional file 2: Table S3, along with the minimal CNV frequency after probe exclusion.

Finally, probes with $p_{Fisher} \leq 0.001$ and a minimum of two disease cases among CNV, duplication, or deletion carriers were retained for assessment through the mirror/U-shaped, duplication-only, or deletion-only model, respectively. The number of probes retained across all models ranged between 0 (sarcoidosis, hyperthyroidism, pituitary dysregulation, rheumatoid arthritis, polycystic kidney disease, and kidney cancer) and 342 (disease burden) (Note S3 – Figure 1C) and correlated with case number of the disease, aligned with the expected gain in power for more frequent diseases (Note S3 – Figure 1D). Number of probes retained according to different models for the main CNV-GWAS are listed in Additional file 2: Table S2. The rationale behind this pre-selection is to reduce computation time without losing any associations, as it is highly unlikely that a genotypic test with $p > 0.001$ would yield a genome-wide significant ($p \leq 7.5 \times 10^{-6}$) logistic regression p-value.

Note S3 – Figure 2. Genomic inflation factor of probe genotypic Fisher tests
(**A**) QQ plots depicting the expected (y-axis) against observed (x-axis) negative logarithm of the genotypic Fisher test's p-values assessing the association strength between the copy-number status of 18,725 probes that passed the CNV frequency filter of ≥ 0.01% and pruning at $r^2 > 0.9999$ and the 60 diseases and disease burden (top stripe). Data points are expected to follow the dark gray line (95% confidence interval as gray shaded area). Phenotypes are ordered by decreasing genomic inflation factor (λ), whose value is indicated in the top left corner. (**B**) Boxplots of λ values across all 61 phenotypes (y-axis) obtained when excluding an increasing percentage (0-80%) of probes with the lowest CNV frequency (x-axis). The red line indicates $\lambda = 1$ (i.e., no inflation).

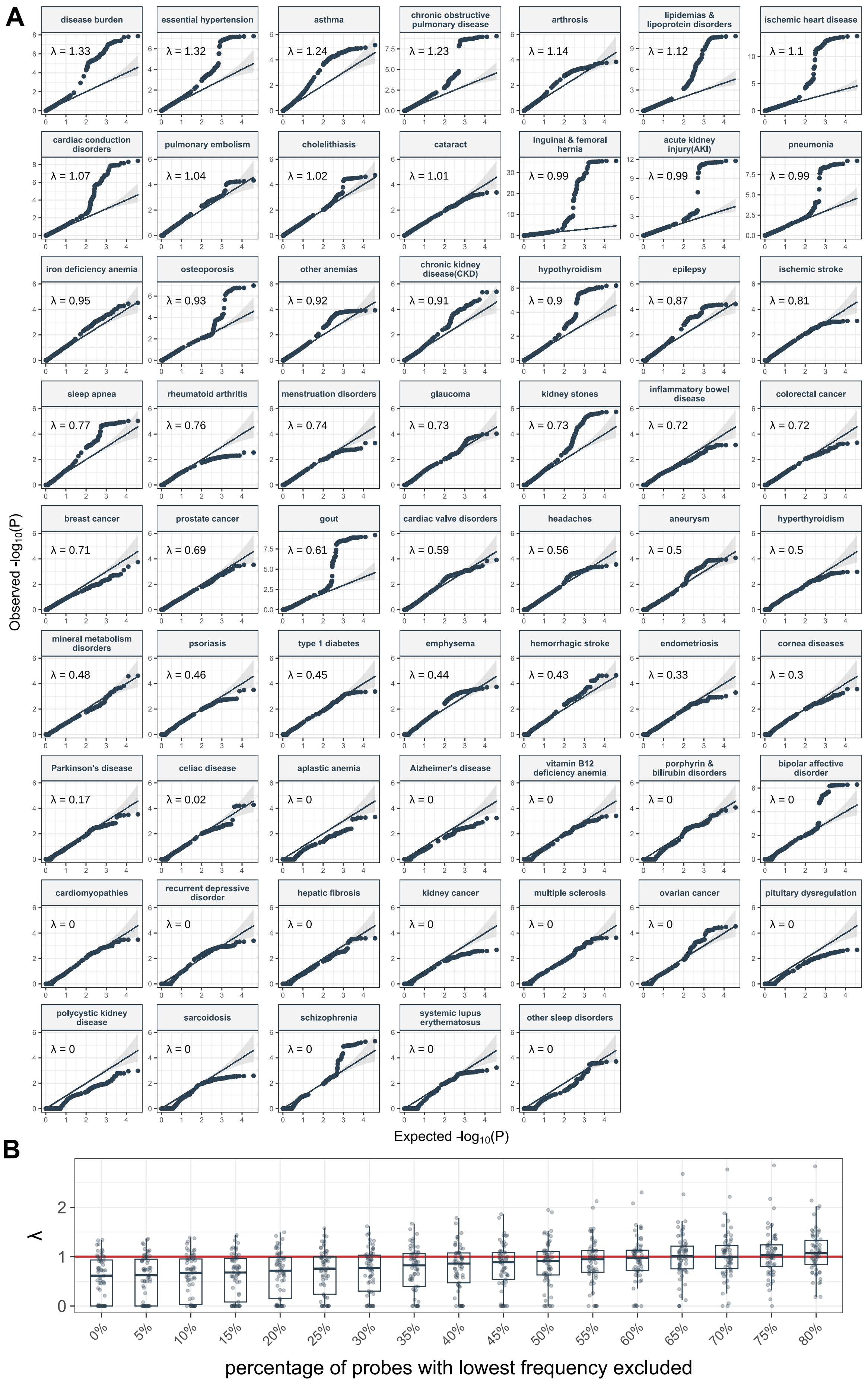
A
disease burden
λ = 1.33
essential hypertension
λ = 1.32
asthma
λ = 1.24
chronic obstructive pulmonary disease
λ = 1.23
arthrosis
λ = 1.14
lipidemias & lipoprotein disorders
λ = 1.12
ischemic heart disease
λ = 1.1
cardiac conduction disorders
λ = 1.07
pulmonary embolism
λ = 1.04
cholelithiasis
λ = 1.02
cataract
λ = 1.01
inguinal & femoral hernia
λ = 0.99
acute kidney injury(AKI)
λ = 0.99
pneumonia
λ = 0.99
iron deficiency anemia
λ = 0.95
osteoporosis
λ = 0.93
other anemias
λ = 0.92
chronic kidney disease(CKD)
λ = 0.91
hypothyroidism
λ = 0.9
epilepsy
λ = 0.87
ischemic stroke
λ = 0.81
sleep apnea
λ = 0.77
rheumatoid arthritis
λ = 0.76
menstruation disorders
λ = 0.74
glaucoma
λ = 0.73
kidney stones
λ = 0.73
inflammatory bowel disease
λ = 0.72
colorectal cancer
λ = 0.72
breast cancer
λ = 0.71
prostate cancer
λ = 0.69
gout
λ = 0.61
cardiac valve disorders
λ = 0.59
headaches
λ = 0.56
aneurysm
λ = 0.5
hyperthyroidism
λ = 0.5
mineral metabolism disorders
λ = 0.48
psoriasis
λ = 0.46
type 1 diabetes
λ = 0.45
emphysema
λ = 0.44
hemorrhagic stroke
λ = 0.43
endometriosis
λ = 0.33
cornea diseases
λ = 0.3
Parkinson's disease
λ = 0.17
celiac disease
λ = 0.02
aplastic anemia
λ = 0
Alzheimer's disease
λ = 0
vitamin B12 deficiency anemia
λ = 0
porphyrin & bilirubin disorders
λ = 0
bipolar affective disorder
λ = 0
cardiomyopathies
λ = 0
recurrent depressive disorder
λ = 0
hepatic fibrosis
λ = 0
kidney cancer
λ = 0
multiple sclerosis
λ = 0
ovarian cancer
λ = 0
pituitary dysregulation
λ = 0
polycystic kidney disease
λ = 0
sarcoidosis
λ = 0
schizophrenia
λ = 0
systemic lupus erythematosus
λ = 0
other sleep disorders
λ = 0
Observed -log10(P)
Expected -log10(P)
B
λ
0%
5%
10%
15%
20%
25%
30%
35%
40%
45%
50%
55%
60%
65%
70%
75%
80%
percentage of probes with lowest frequency excluded

Note S4: Post-CNV-GWAS summary statistics processing

Summary statistics harmonization

Given the encoding of CNVs in PLINK (Note S1), we want to obtain the effect of carrying an additional "T" for the mirror (i.e., effect of increasing number of copies), duplication-only (i.e., effect of the duplication), and the deletion-only (i.e., effect of the deletion) models. PLINK selects the effect allele ("A1") as the minor allele, so that depending on the deletion and duplication frequencies, it will report the effect of "A" or "T". In the former case, odds ratios (OR) and their 95% confidence interval (CI) were harmonized to "T", i.e., $OR_T = \frac{1}{OR_A}$ and $CI_T = e^{\log(OR_T) \pm 1.96 * SE_{\log(OR_A)}}$, respectively. Because we use the `hetonly` modifier for the U-shape model, PLINK systematically reports the effect of being "AT", i.e., copy-neutral. To instead obtain the effect of having a CNV, the same transformation as described above was applied to all probes. For the disease burden, which was assessed through linear regression, $\beta_T = -1 * \beta_A$ was applied when PLINK reported the effect of "A". Similarly, the confidence interval was multiplied by -1 and inverted, i.e., the lower bound becomes the upper bound and vice-versa.

Conditional analysis

Because of the high correlation between the copy-number state of tested probes, it is important to determine the number of independent CNV-disease associations identified. Genome-wide significant associations ($p \leq 7.5 \times 10^{-6}$; *Genome-wide significance threshold*) were pruned at $r^2 > 0.8$ (`--indep-pairwise 3000 500 0.8` PLINK v2.0). As PLINK preferentially keeps probes with higher nonmajor allele frequencies, we inputted a scaled negative logarithm of association p-value as frequency (`--read-freq` PLINK v2.0) to instead prioritize probes with the strongest association p-value. For the U-shape model, pruning was performed using custom code by extracting probes from $PLINK_{CNV}$ and re-coding them to match U-shape numerical encoding. Number of independent signals per disease was determined by stepwise conditional analysis. Briefly, for each disease and association model, the CNV genotype of the lead probe (i.e., probe with the most significant association p-value at each iteration; encoding numerically as in Note S1) was included along selected covariates in the logistic regression model and association studies were conducted anew. This process was repeated in an iterative fashion, always including the next lead probe as an additional covariate, until no probes passed the genome-wide significant threshold.

Note S5: Estonian Biobank replication

EstBB disease definition

Disease cases and disease burden were defined using the same inclusion and exclusion criteria as for the UKBB, with the notable exceptions of excluding Z12 (routine preventive screens for cancer) and D22-23 (benign skin lesions) subcodes from the exclusion list of cancer traits as due to differences in recording practices, these were much more frequent in the EstBB than in the UKBB, strongly reducing the number of controls. Furthermore, as there are no self-reported diagnoses available in the EstBB, the latter could not be used as an exclusion criterion for disease definition in the EstBB.

CNV calling and sample selection

Autosomal CNVs were called from Illumina Global Screening Array genotype data for 193,844 individuals that survived general quality control and had i) matching genotype-phenotype identifiers, ii) matching inferred versus reported sex, iii) a SNP-call rate ≥ 98%, iv) were of European ancestry (i.e., Europe (East), Europe (South West), Europe (North West), Finland, and Italy assignments from the `bigsnpr` R package function `snp_ancestry_summary()` [6]), and v) were included in the EstBB SNP imputation pipeline. CNV outlier samples based on genotyping plate or extreme CNV profile, as well as individuals reporting blood malignancies were excluded, using the same criteria as for the UKBB (Notes S1 and S2). High confidence CNV calls (i.e., with quality score (QS) value of: |QS| > 0.5) of the 156,254 remaining individuals were encoded into three PLINK binary file sets, following the procedure described for the UKBB (Note S1).

EstBB replication analysis

Related individuals with available CNV calls were pruned (`--make-king-table --king-table-filter 0.0884 --geno 0.05 --maf 0.01` PLINK v2.0; kinship coefficient > 0.0884 corresponding to <3rd degree relatedness), prioritizing individuals whose disease status was least often missing, leaving 90,211 unrelated samples for the replication study. Disease-relevant covariates were selected among sex, year of birth, genotyping batch (1-11), and PC1-20. For each of the unique 68 autosomal CNVR-disease association signals identified in the UKBB, we identified EstBB probes that were overlapping the CNVR's genomic coordinates. Probes with an EstBB CNV, duplication, or deletion frequency ≥ 0.01%, were retained, depending on whether the mirror/U-shape, duplication-only, or deletion-only was the best UKBB model, respectively, and 11 signals were excluded due to null/low CNV frequency for all probes in the CNVR. Association studies were performed on remaining probes using disease-specific covariates and the best UKBB model, following the previously described

procedure. Probes for which the regression failed to converge were discarded, leading to the exclusion of 8 signals for which all regressions failed. Summary statistics of the EstBB probe with the closest genomic location to the lead UKBB probe were retrieved for the remaining 49 signals, setting the replication threshold for significance at $p \leq 0.05/49 = 1.0 \times 10^{-3}$. P-values were adjusted to account for directional concordance with UKBB effects by rewarding and penalizing signals with matching and non-matching effect size signs, respectively. Specifically, one-sided p-values were obtained as $p_{new} = \frac{p_{old}}{2}$ and $p_{new} = 1 - (\frac{p_{old}}{2})$ for 35 concordant and 14 non-concordant signals, respectively. One-sided binomial tests (`binom.test()`) were used to assess enrichment of observed versus expected significant replications at various thresholds (α = 0.1 to 0.005 by steps of 0.005), with the R function arguments: x the number of observed signals at α, n the number of testable signals (i.e., 49), and p the expected probability of signals meeting α (i.e., α).

Note S6: Subgrouping of CNV carriers

When analyzing complex CNVRs (i.e., 16p13.11, 22q11.2, 15q13), CNV carriers were split into subgroups based on visual inspection of breakpoints and segmental duplications overlapping the region. Criteria below were used to define groups (Note S6 – Table 1). CNVs not matching any of the groups are referred to as “atypical” CNVs.

CNVR	group	chr	min. start [bp]	max. start [bp]	min. end [bp]	max. end [bp]
**16p13.11** (Figure 5)	cat1	16	-	15,000,000	16,250,000	16,750,000
	cat2	16	15,000,000	15,200,000	16,250,000	16,750,000
	cat3	16	15,200,000	15,800,000	16,250,000	16,750,000
	cat4	16	-	15,800,000	17,500,000	-
	cat5*	16	16,242,785	-	-	16,317,379
**15q13** (Figure 6)	BP4-5	15	30,250,000	31,250,000	32,300,000	33,100,000
	D-CHRNA7-BP5	15	31,700,000	32,300,000	32,300,000	33,100,000
**22q11.2** (Note S10)	LCR A-D	22	18,500,000	19,200,000	21,250,000	21,900,000
	LCR A-B	22	18,500,000	19,200,000	20,250,000	20,600,000
	LCR B-D	22	20,000,000	20,850,000	21,250,000	21,900,000
	LCR C-D	22	21,000,000	21,150,000	21,250,000	21,900,000

Note S6 – Table 1. CNV carrier subgrouping
Selection criteria for different CNV carrier subgroups considered for analyzed CNV regions (related Figure/Supplemental Note in parenthesis). Minimum and maximum start and end positions reflect the range in which the CNV breakpoints are to be for a given CNV to be assigned to a subgroup. “-” indicates open end. *For 16p13.11 cat5 CNVs, coordinates correspond to the coordinates of *ABCC6*. All positions are in hg19/GRCh37.

Note S7: *BRCA1* deletion association with ovarian and other female cancers

Methods

Medical history of female *BRCA1* deletion carriers is based on #41270 (*diagnosis – ICD10*) and age at diagnosis was calculated as previously described (*Case-control definition and age-at-disease onset calculation*). Relevant and prevalent diagnoses were manually selected for display. For the hereditary breast and ovarian cancer (HBOC) prevalence and time-to-event analysis, we considered C50 (malignant neoplasm of breast), C53 (malignant neoplasm of cervix uteri), C54 (malignant neoplasm of corpus uteri), C55 (malignant neoplasm of uterus, part unspecified), C56 (malignant neoplasm of ovary), and C57 (malignant neoplasm of other unspecified female genital organs) ICD-10 diagnoses on the inclusion list and used the same exclusion list as for ovarian cancer. Duplications and low-quality CNVs ($|QS| \leq 0.5$), as well as male individuals were excluded from the analyses. Difference in prevalence was assessed with a two-sided Fisher test. Time-to-event analysis was performed as previously described (*Statistical confidence tiers*) to estimate the effect of the *BRCA1* deletion, using age, age^2, array, and PC1-40 as covariates.

Results

Two out of 12 female *BRCA1* deletion carriers were diagnosed with ovarian cancer (chr17:41,197,733-41,276,111; $OR_{del} = 284.3$; 95%-CI [24.6; 3290.8]; $p = 6.1 \times 10^{-6}$; Note S7 – Figure 1A). *BRCA1* [MIM: 113705] is a tumor suppressor gene whose loss-of-function (LoF) represents a major genetic risk factor for the development of HBOC [MIM: 604370] [7]. Exploring the clinical records of the 12 deletion carriers, we found five diagnoses of breast cancer (a trait assessed by CNV-GWAS but that did not yield a GW-significant association), one of endometrial cancer, and one of Fallopian tube cancer, so that eight carriers (67%) had received a HBOC diagnosis (Note S7 – Figure 1B). Not only was prevalence of HBOC higher among *BRCA1* deletion carriers ($OR_{Fisher} = 31.0$; $p = 1.1 \times 10^{-6}$), but disease onset was earlier (HR = 17.0; $p = 1.3 \times 10^{-15}$; Note S7 – Figure 1C). Among the four carriers with no HBOC, two had received cancer prophylactic surgery, *de facto* reducing the penetrance of the deletion. Surgeries were likely carried out based on family history of HBOC, which was reported for 6 carriers (50%), suggesting that these deletions are inherited. We did not observe higher prevalence of other cancer types (Note S7 – Figure 1B).

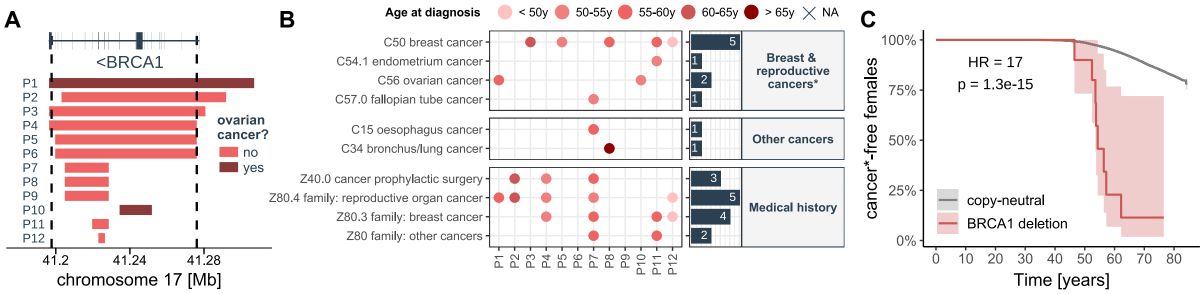

Note S7 – Figure 1. *BRCA1* deletion association with ovarian and other female cancers

(**A**) Genomic coordinates of the 12 females (P1-12; y-axis) carrying a *BRCA1* deletion (CNVR delimited by vertical dashed lines), colored according to ovarian cancer diagnosis. (**B**) Left: Cancer and related family/personal diagnoses received by individuals in (A). Color indicates age at diagnosis. Right: Counts per ICD-10 code. (**C**) Kaplan-Meier curve depicting the percentage, with 95% confidence interval, of females free of female-specific cancers over time among copy-neutral and *BRCA1* deletion carriers. Hazard ratio (HR) and p-value for the *BRCA1* deletion are given (CoxPH model).

Note S8: *LDLR* deletion association with ischemic heart disease

Methods

Medical history of low-density lipoprotein (LDL) receptor (*LDLR*) deletion carriers is based on #41270 (*diagnosis – ICD10*) and age at diagnosis was calculated as previously described (*Case-control definition and age-at-disease onset calculation*). Drug usage data originates from #20003 (*treatment/medication code*). The list of considered hypolipidemic agents and antihypertensive/antianginal drugs (Note S8 – Table 1) was based on: https://www.drugs.com/ (accessed: 29/09/2022). A minimum of 3 individuals was required for a code/drug to be displayed.

Category	Description	UKBB_code
**statins**	atorvastatin	1141146234
	lipitor 10mg tablet	1141146138
	fluvastatin	1140888594
	lescol 20mg capsule	1140864592
	pravastatin	1140888648
	rosuvastatin	1141192410
	crestor 10mg tablet	1141192414
	simvastatin	1140861958
	zocor 10mg tablet	1140881748
	zocor heart-pro 10mg tablet	1141200040
	eptastatin	1140910632
	velastatin	1140910654
**cholesterol absorption inhibitors**	ezetimibe	1141192736
	ezetrol 10mg tablet	1141192740
**fibrates**	fenofibrate	1140861954
	gemfibrozil	1140861856
	gemfibrozil product	1141157262
	lopid 300 capsule	1140861858
	clofibrate	1140861944
	bezafibrate product	1141157260
	bezafibrate	1140861924
	bezalip 200mg tablet	1140861926
	bezalip-mono 400mg m/r tablet	1140861928
**bile acid sequestrants**	cholestyramine+aspartame 4g/sachet powder	1140861942
	cholestyramine	1140865576
	cholestyramine product	1141157416
	questran 4g/sachet powder	1140861936
	colestipol	1140888590
	colestid 5g/sachet granules	1140861848
**cardioselective beta-blocker**	atenolol	1140866738
	bisoprolol	1140879760
	cardicor 1.25mg tablet	1141171152
**ACE inhibitor**	ramipril	1140860806
	perindopril	1140888560
	lisinopril	1140860696

Note S8 – Table 1. Considered drugs with UK Biobank encoding
Drugs from #20003 considered in the displayed drug categories in Note S8 - Figure 1B. UK Biobank encoding is provided in the last column.

For prevalence and time-to-event analysis, only E78.0 (pure hypercholesterolemia) was considered on the inclusion list; the same exclusion list as for lipidemia was used. Duplications

and low-quality CNVs (|QS| ≤ 0.5) were excluded from analyses. Difference in prevalence was assessed with a two-sided Fisher test. Time-to-event analysis was performed as previously described (*Statistical confidence tiers*) to estimate the effect of the *LDLR* deletion, using sex, age, age^2, array, and PC1-40 as covariates.

LDL cholesterol measurements were available for seven *LDLR* deletion carriers in #42040 (*GP clinical event records*). LDL levels of earliest measurement on record (primary care) were compared to LDL levels from standardized blood biochemistry measurement (#30780) taken at assessment (#53) using a one-sided paired t-test. P12 was excluded as blood biochemistry LDL levels preluded the first primary care measurement. Based on #42039 (*GP prescription records*), P5 and P13 were identified as being prescribed statins by their general practitioner despite no record of statin usage in #20003.

Results

High abundance of *Alu* repeats make the *LDLR* gene [MIM: 606945] susceptible to CNVs [8]. We found that deletion of exon 2-6 increased risk for ischemic heart disease (chr19:11,210,904-11,218,188; OR_{del} = 31.2; 95%-CI [7.1; 137.8]; $p = 5.6 \times 10^{-6}$) in a BMI-independent fashion. The condition was present in 8 of 14 deletion carriers (Note S8 – Figure 1A). Heterozygous - and less frequently homozygous – mutations in *LDLR* represent the main genetic etiology for familial hypercholesterolemia [9], which is characterized by elevated LDL cholesterol and predisposition for adverse cardiovascular outcomes [10]. Previously identified in clinical studies of familial hypercholesterolemia [11], the CNVR implicated by our analysis specifically encompasses the ligand-binding domain of *LDLR* [9]. Confirming widespread prevalence and family history (43%) of cardiovascular diseases (Note S8 – Figure 1B), medical records of deletion carriers further revealed higher prevalence (OR_{Fisher} = 11.6; $p = 7.9 \times 10^{-5}$) and earlier onset (HR = 5.8; $p = 1.4 \times 10^{-7}$; Note S8 – Figure 1C) of pure hypercholesterolemia (E78.0), a code included in our lipidemia definition but that did not yield a signal pick-up by the CNV-GWAS. As we previously did not find the CNVR to associate with standardized blood biochemistry LDL levels [1], we hypothesized that the latter were lowered by hypolipidemic agents (Note S8 – Table 1). Ten (71%) deletion carriers were on statins and six (43%) were additionally using cholesterol absorption inhibitors, while the remaining four did not receive a dyslipidemia or ischemic heart disease diagnosis and harbored smaller deletions (i.e., P12-14; Note S8 – Figure 1A-B). We concluded that drugs likely masked genetically determined LDL levels, as shown by higher LDL levels in the first primary care measurement on record, measured prior to the standardized LDL measurement ($p_{t\text{-}test}$ = 0.03; Note S8 – Figure 1D). Despite this, the recommended target of ≤ 1.8 mmol/L for high-risk individuals [12] was never met.

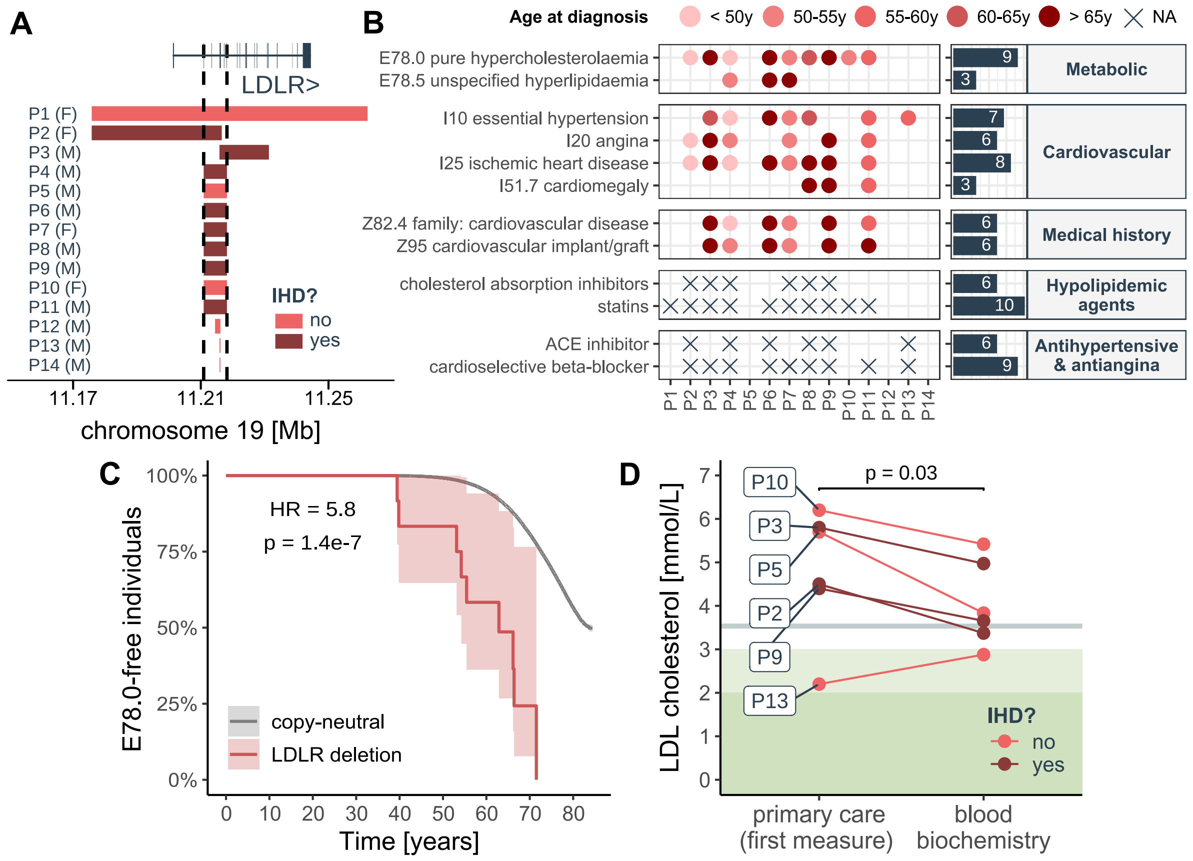

Note S8 – Figure 1. *LDLR* deletion association with ischemic heart disease

(**A**) Genomic coordinates of the 14 individuals (P1-14; y-axis) carrying an *LDLR* deletion (CNVR delimited by vertical dashed lines), colored according to ischemic heart disease (IHD) diagnosis. Sex of the individuals is indicated, with (M) corresponding to male and (F) to female (**B**) Left: Medical conditions and family/personal diagnoses and medication received by ≥ 3 *LDLR* deletion carriers in (A). Color indicates age at diagnosis. Right: Counts per ICD-10 code. (**C**) Kaplan-Meier curve depicting the percentage, with 95% confidence interval, of individuals free of pure hypercholesterolemia (E78.0) among copy-neutral and *LDLR* deletion carriers. Hazard ratio (HR) and p-value for the *LDLR* deletion are given (CoxPH model). (**D**) Low density lipoprotein (LDL)-cholesterol levels (y-axis) from primary care data (first available measurement) and blood biochemistry (average over instances) for six deletion carriers in (A) with at least one antecedent primary care LDL-cholesterol measurement, colored according to IHD diagnosis. P-value compares the two data sources (paired one-sided t-test). Gray horizontal line represents median LDL-cholesterol value (from blood biochemistry) in non-carriers. Light and darker green background represent recommended target values for low (≤ 3 mmol/L) and high (≤ 1.8 mmol/L) risk individuals, respectively.

Note S9: 16p12.2 deletion associations

Results

The blood pressure-increasing 16p12.2 deletion (chr16:21,946,523-22,440,319) [1,13] increased risk for hypertension (OR_{del} = 2.7; 95%-CI [1.9; 3.8]; $p = 1.3 \times 10^{-8}$) and cardiac conduction disorders (OR_{del} = 3.3; 95%-CI [2.2; 4.9]; $p = 1.1 \times 10^{-8}$), suggesting a role in cardiovascular health (Note S9 – Figure 1A-D). Primarily associated with developmental delay and intellectual disability [14,15] – proxied by decreased fluid intelligence ($p_{t\text{-test}} = 8.7 \times 10^{-5}$) and income ($p_{t\text{-test}} = 1.4 \times 10^{-12}$) in the UKBB (Note S9 – Figure 1E-F) – cardiac malformations are reported in ~38% of clinically ascertained cases [16]. Among 193 UKBB deletion carriers, two (1%) had congenital insufficiency of the aortic valve (Q23.1), corresponding to a higher but not significantly different prevalence of cardiovascular malformations (Q20-28) than in copy-neutral individuals (OR_{Fisher} = 2.1; p = 0.251). The deletion also associated with increased risk for pneumonia (OR_{del} = 3.0; 95%-CI [1.9; 4.6]; $p = 5.4 \times 10^{-7}$) and decreased forced vital capacity [1] (Note S9 – Figure 1G-H) and peak expiratory flow [13].

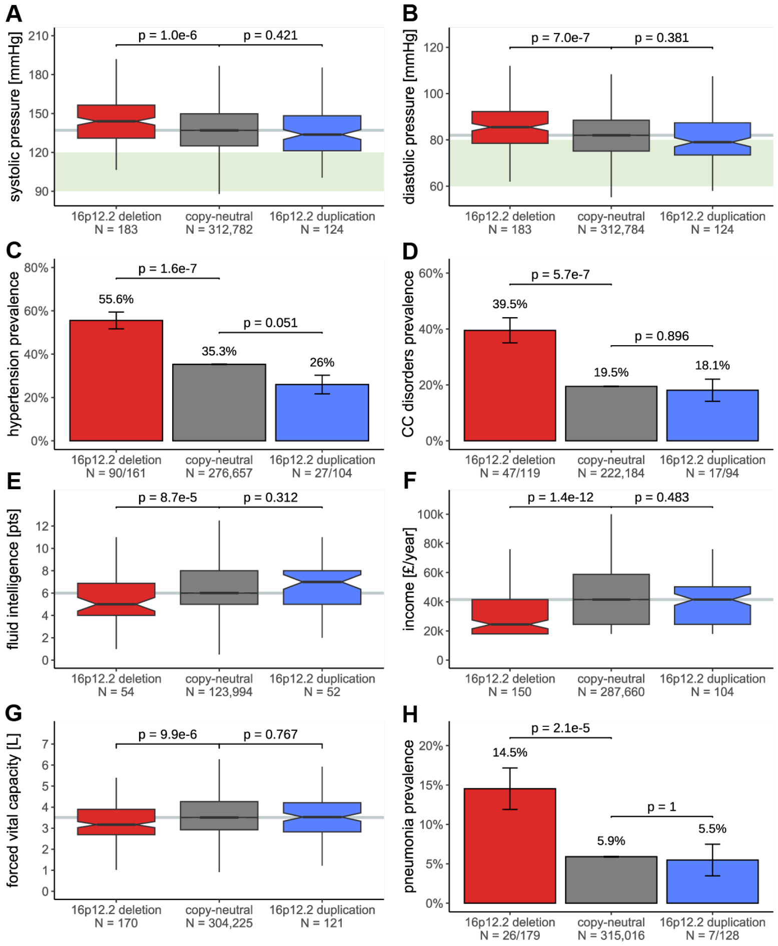

Note S9 – Figure 1. 16p12.2 CNV region
Boxplots of (**A**) systolic (UKBB field #4080) and (**B**) diastolic (#4079) blood pressure according to 16p12.2 copy-number (CN). Green background represents optimal blood pressure (systolic: 90-120 mmHg; diastolic: 60-80 mmHg). Bar plots of (**C**) essential hypertension and (**D**) cardiac conduction (CC) disorders prevalence according to 16p12.2 CN. Boxplots of (**E**) fluid intelligence score (#20016; maximum = 13 points), (**F**) average yearly total household income before taxes (#738: ≤ £18k to £18k; £18k-30.9 to £24.5k; £31k-51.9 to £41.5k; £52k-100k to £76k; ≥ £100k to £100k), and (**G**) forced vital capacity (#3062) according to 16p12.2 CN, shown as boxplots. (**H**) Pneumonia prevalence according to 16p12.2 CN. For boxplots, outliers are not shown; p-values compare deletion and duplication carriers to copy-neutral individuals (two-sided t-test); gray horizontal line represents median among copy-neutral individuals; N indicates sample sizes. For bar plots, error bars represent ± the standard error; p-values compare prevalence among deletion and duplication carriers to the one in copy-neutral individuals (two-sided fisher test); N indicates cases count on sample size.

Note S10: 22q11.2 CNV associations

Results

The proximal 22q11.2 region, previously linked to DiGeorge [MIM: 188400] and velocardiofacial [MIM: 192430] syndromes, harbors four low-copy repeats (LCR; labeled A-to-D) [17]. Building on evidence of complex association patterns within this CNVR [18], we report novel associations between CNVs spanning LCR A-D and ischemic heart disease (IHD; chr22:19,024,651-21,463,545; $OR_{U\text{-}shape}$ = 2.1; 95%-CI [1.6; 2.8]; p = 1.5 x 10^{-7}), LCR B-D and aneurysm (chr22:20,708,685-21,460,008; OR_{del} = 41.8; 95%-CI [10.0; 175.1]; p = 3.2 x 10^{-7}), and LCR A-C and headaches (chr22:19,024,651-21,110,240; $OR_{mirror.}$ = 3.7; 95%-CI [2.1; 6.5]; p = 4.8 x 10^{-6}) (Note S10 – Figure 1A). Based on 3 LCR B-D deletion carriers with aneurysm, this corresponds to a 22-times higher prevalence than in copy-neutral individuals (Note S10 – Figure 1B). Association with IHD is better powered, with a prevalence of 12%, 21%, 16%, and 20% among copy-neutral individuals and carriers of LCR C-D, B-D, and A-D CNVs, respectively (Note S10 – Figure 1C). Unlike the association with aneurysm, association with IHD was lost upon adjustment for body mass index (BMI). This suggests that IHD risk is driven by increased adiposity which scales with the amount of affected genetic content, supporting the presence of multiple genetic driver(s). Collectively, our data indicates that altered 22q11.2 dosage can result in a spectrum of cardiovascular afflictions of various degrees of severity, ranging from well-described congenital malformation [17,19] to adult-onset aneurysm or IHD.

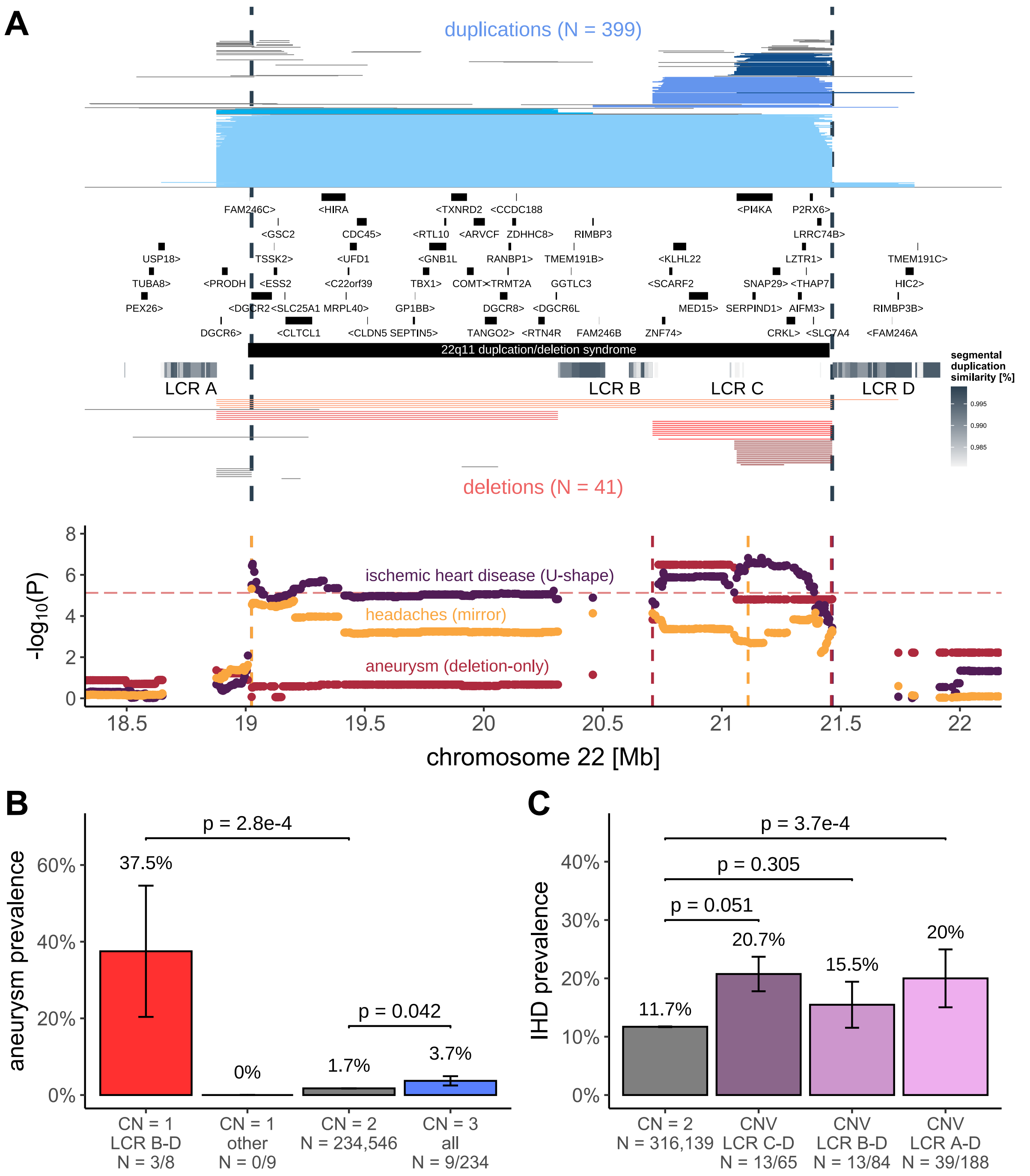

Note S10 – Figure 1. 22q11.2 CNV region

(**A**) 22q11.2 genetic landscape. Top: Coordinates of duplications (shades of blue; top) and deletions (shades of red; bottom) overlapping the maximal CNV region (CNVR delimited by vertical dashed lines) associated with ischemic heart disease (IHD), headaches, and aneurysm. CNVs are divided and colored according to four groups to reflect breakpoints at low-copy repeats (LCRs) spanning the region: A-D, A-B, B-D, C-D, with atypical CNVs in gray (Note S6). LCRs are composed of segmental duplications, represented as a gray gradient proportional to the degree of similarity. Genomic coordinates of genes and DECIPHER GD are displayed. Bottom: Negative logarithm of association p-values of CNVs (best model in parenthesis) with IHD, headaches, and aneurysm. Disease-specific CNVRs are shown with colored vertical dashed lines. Red horizontal dashed line represents the genome-wide threshold for significance for CNV-GWAS ($p \leq 7.5 \times 10^{-6}$). (**B**) Prevalence of aneurysm according to 22q11.2 copy-number (CN) and CNV group (A). P-values compare deletion (CN = 1) and duplication (CN = 3) carriers from various groups (other = A-D, A-B, C-D; all = A-D, A-B, B-D, C-D) to copy-neutral (CN = 2) individuals (two-sided Fisher test). (**C**) Prevalence of IHD according to CNV groups (A). P-values compare IHD prevalence among individuals carrying a CNV (duplication or deletion) spanning LCR C-D, B-D, or A-D to copy-neutral (CN = 2) individuals (two-sided Fisher test). (B, C) Error bars represent ± standard error; number of cases and sample sizes are indicated (N = cases/sample size).

REFERENCES

1. Auwerx C, Lepamets M, Sadler MC, Patxot M, Stojanov M, Baud D, et al. The individual and global impact of copy-number variants on complex human traits. Am J Hum Genet. 2022;109:647–68.
2. Manichaikul A, Mychaleckyj JC, Rich SS, Daly K, Sale M, Chen W-M, et al. Robust relationship inference in genome-wide association studies. Bioinformatics. 2010;26:2867–73.
3. Bycroft C, Freeman C, Petkova D, Band G, Elliott LT, Sharp K, et al. The UK Biobank resource with deep phenotyping and genomic data. Nature. 2018;562:203–9.
4. Wu P, Gifford A, Meng X, Li X, Campbell H, Varley T, et al. Mapping ICD-10 and ICD-10-CM Codes to Phecodes: Workflow Development and Initial Evaluation. JMIR Med Inform. 2019;7:e14325.
5. Yang J, Weedon MN, Purcell S, Lettre G, Estrada K, Willer CJ, et al. Genomic inflation factors under polygenic inheritance. European Journal of Human Genetics. 2011;19:807–12.
6. Prive F. Using the UK Biobank as a global reference of worldwide populations: application to measuring ancestry diversity from GWAS summary statistics. Bioinformatics. 2022;38:3477–80.
7. Miki Y, Swensen J, Shattuck-Eidens D, Futreal PA, Harshman K, Tavtigian S, et al. A strong candidate for the breast and ovarian cancer susceptibility gene BRCA1. Science (1979). 1994;266:66–71.
8. Iacocca MA, Hegele RA. Role of DNA copy number variation in dyslipidemias. Curr Opin Lipidol. 2018;29:125–32.
9. Hobbs HH, Russell DW, Brown MS, Goldstein JL. The LDL receptor locus in familial hypercholesterolemia: mutational analysis of a membrane protein. Annu Rev Genet. 1990;24:133–70.
10. Defesche JC, Gidding SS, Harada-Shiba M, Hegele RA, Santos RD, Wierzbicki AS. Familial hypercholesterolaemia. Nat Rev Dis Primers. 2017;3:17093.
11. Iacocca MA, Wang J, Dron JS, Robinson JF, McIntyre AD, Cao H, et al. Use of next-generation sequencing to detect LDLR gene copy number variation in familial hypercholesterolemia. J Lipid Res. 2017;58:2202–9.
12. Mach F, Baigent C, Catapano AL, Koskinas KC, Casula M, Badimon L, et al. 2019 ESC/EAS Guidelines for the management of dyslipidaemias: lipid modification to reduce cardiovascular riskThe Task Force for the management of dyslipidaemias of the European Society of Cardiology (ESC) and European Atherosclerosis Society (EAS). Eur Heart J. 2020;41:111–88.
13. Owen D, Bracher-Smith M, Kendall KM, Rees E, Einon M, Escott-Price V, et al. Effects of pathogenic CNVs on physical traits in participants of the UK Biobank. BMC Genomics. 2018;19:1–9.
14. Girirajan S, Rosenfeld JA, Cooper GM, Antonacci F, Siswara P, Itsara A, et al. A recurrent 16p12.1 microdeletion supports a two-hit model for severe developmental delay. Nat Genet. 2010;42:203–9.
15. Stefansson H, Meyer-Lindenberg A, Steinberg S, Magnusdottir B, Morgen K, Arnarsdottir S, et al. CNVs conferring risk of autism or schizophrenia affect cognition in controls. Nature. 2013;505:361–6.
16. Girirajan S, Pizzo L, Moeschler J, Rosenfeld J. 16p12.2 Recurrent Deletion. GeneReviews® [Internet]. 2018 [cited 2023 Apr 4]; Available from: https://www.ncbi.nlm.nih.gov/books/NBK274565/
17. McDonald-McGinn DM, Sullivan KE, Marino B, Philip N, Swillen A, Vorstman JAS, et al. 22q11.2 deletion syndrome. Nat Rev Dis Primers. 2015;1:1–19.
18. Zamariolli M, Auwerx C, Sadler MC, Graaf A Van Der, Lepik K, Schoeler T, et al. The impact of 22q11.2 copy-number variants on human traits in the general population. Am J Hum Genet. 2023;110:300–13.
19. Bartik LE, Hughes SS, Tracy M, Feldt MM, Zhang L, Arganbright J, et al. 22q11.2 duplications: Expanding the clinical presentation. Am J Med Genet A. 2022;188:779–87.
