# Supplementary material for: Rare copy-number variants as modulators of common disease susceptibility
Source: Genome Med. 2024 Jan 8;16:5. doi: 10.1186/s13073-023-01265-5 (PMC10773105; doi:10.1186/s13073-023-01265-5)
Supplement: Supplementary file 2 — Additional file 2: Supplemental Figures. Figure S1. Case-control distribution in the UK and Estonian Biobanks. Figure S2. BMI adjustment for possibly confounded CNV-disease associations. Figure S3. Constraint analysis of disease associated CNV regions. Figure S4. Total, corrected, and subset burden analysis. [file 13073_2023_1265_MOESM2_ESM.pdf]

Additional file 2: Supplemental Figures

Rare copy-number variants as modulators of common disease susceptibility

Chiara Auwerx[1,2,3,4,*], Maarja Jõeloo[5,6], Marie C. Sadler[2,3,4], Nicolò Tesio[1], Sven Ojavee[2,3], Charlie J. Clark[1], Reedik Mägi[6], Estonian Biobank Research Team[6,§], Alexandre Reymond[1,#,*] & Zoltán Kutalik[2,3,4,#,*]

[1] Center for Integrative Genomics, University of Lausanne, Lausanne, Switzerland
[2] Department of Computational Biology, University of Lausanne, Lausanne, Switzerland
[3] Swiss Institute of Bioinformatics, Lausanne, Switzerland
[4] University Center for Primary Care and Public Health, Lausanne, Switzerland
[5] Institute of Molecular and Cell Biology, University of Tartu, Tartu 51010, Estonia
[6] Estonian Genome Centre, Institute of Genomics, University of Tartu, Tartu 51010, Estonia
[§] Estonian Biobank Research Team: Tõnu Esko, Andres Metspalu, Lili Milani, Reedik Mägi, Mari Nelis
[#] These authors jointly supervised this work.

*** Correspondence:**
Chiara Auwerx: chiara.auwerx@unil.ch;
Alexandre Reymond : alexandre.reymond@unil.ch;
Zoltán Kutalik: zoltan.kutalik@unil.ch.

Figure S1. Case-control distribution in the UK and Estonian Biobanks.
Figure S2. BMI adjustment for possibly confounded CNV-disease associations.
Figure S3. Constraint analysis of disease associated CNV regions.
Figure S4. Total, corrected, and subset burden analysis.

Figure S1: Case-control distribution in the UK and Estonian Biobanks

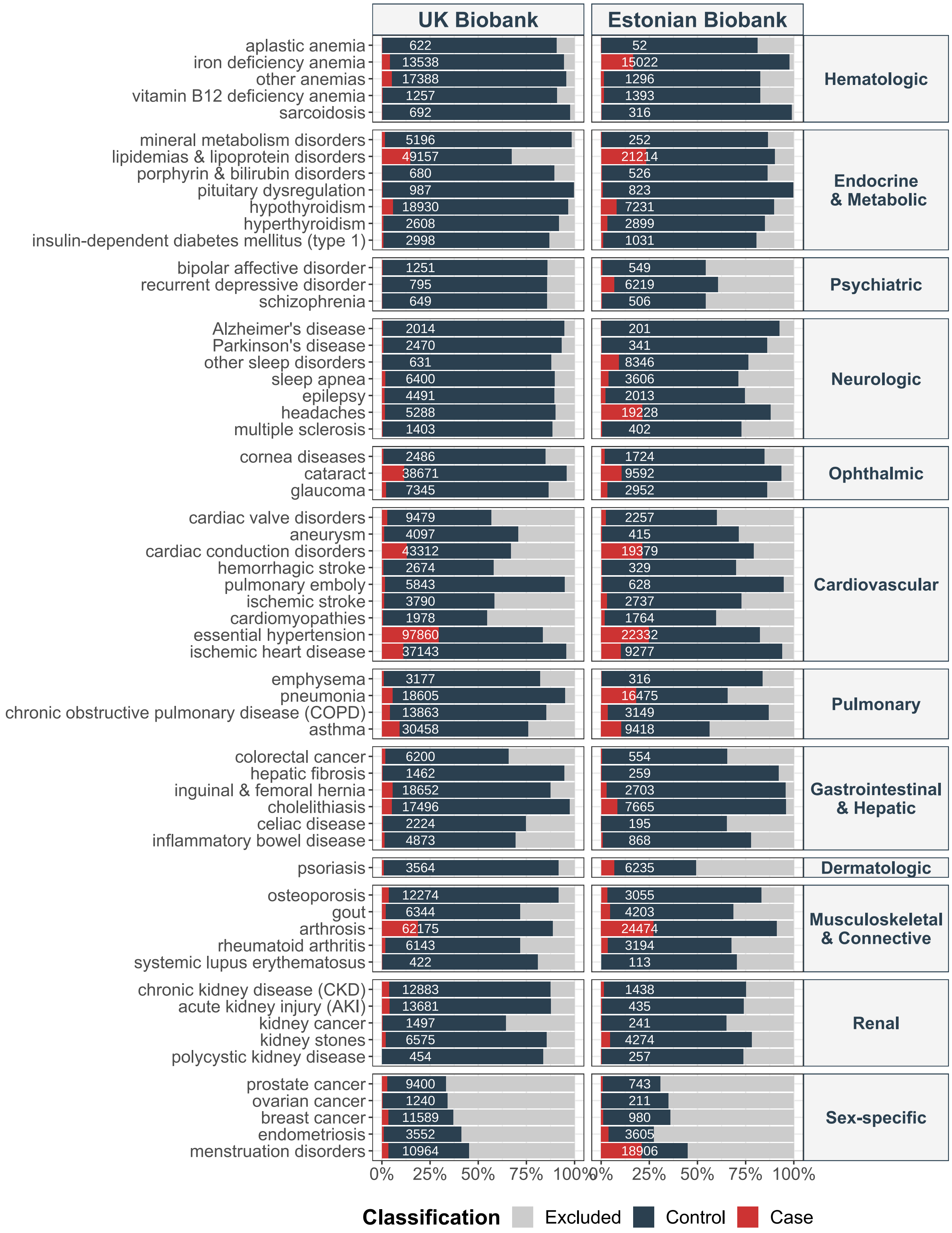

Figure S1. Case-control distribution in the UK and Estonian Biobanks
UK Biobank (left) and Estonian Biobank (right) percent stacked bar chart (x-axis) of cases (red), controls (dark gray), and excluded (gray) individuals, for each of the 60 assessed diseases (y-axis; left) categorized according to their ICD-10 chapter (y-axis; right). Case count is indicated in white on each bar.

Figure S2: BMI adjustment for possibly confounded CNV-disease associations

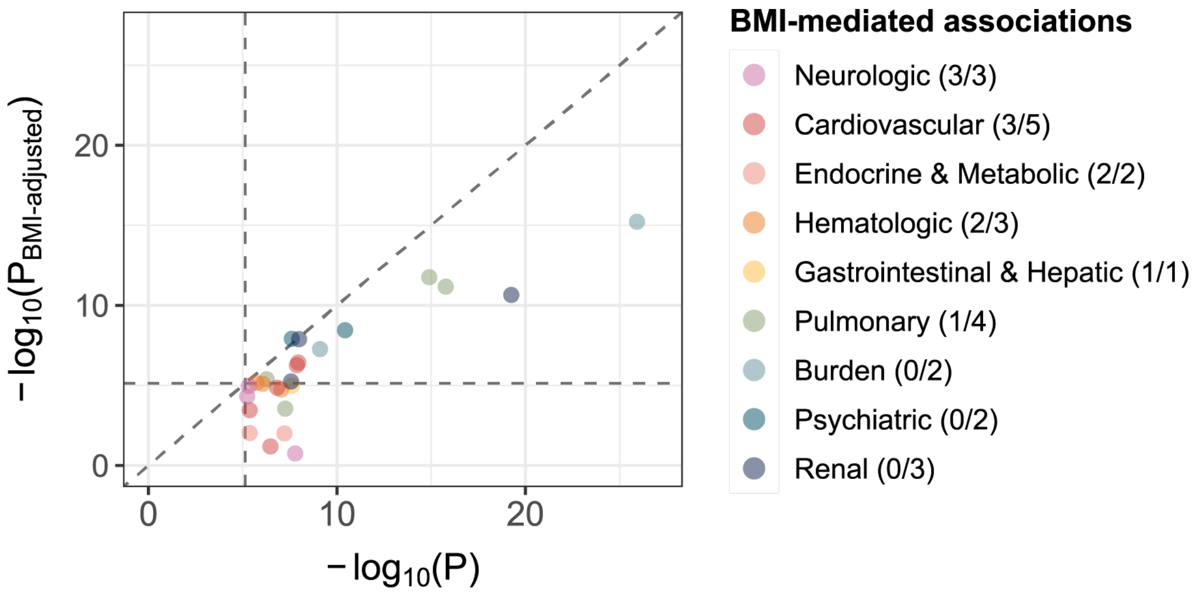

Figure S2. BMI adjustment for possibly confounded CNV-disease associations.
Negative logarithm of CNV-disease association p-value with (y-axis) and without (x-axis) adjustment for body mass index (BMI) for the 25 CNV-GWAS signals potentially confounded by the latter. The horizontal and vertical dashed lines represent the genome-wide significance threshold at $p \leq 7.5 \times 10^{-6}$; the diagonal dashed line represents the identity line. Associations are colored by ICD-10 chapter, with the number of associations that fail to reach genome-wide significance upon adjustment for BMI indicated in parenthesis.

Figure S3: Constraint analysis of disease associated CNV regions

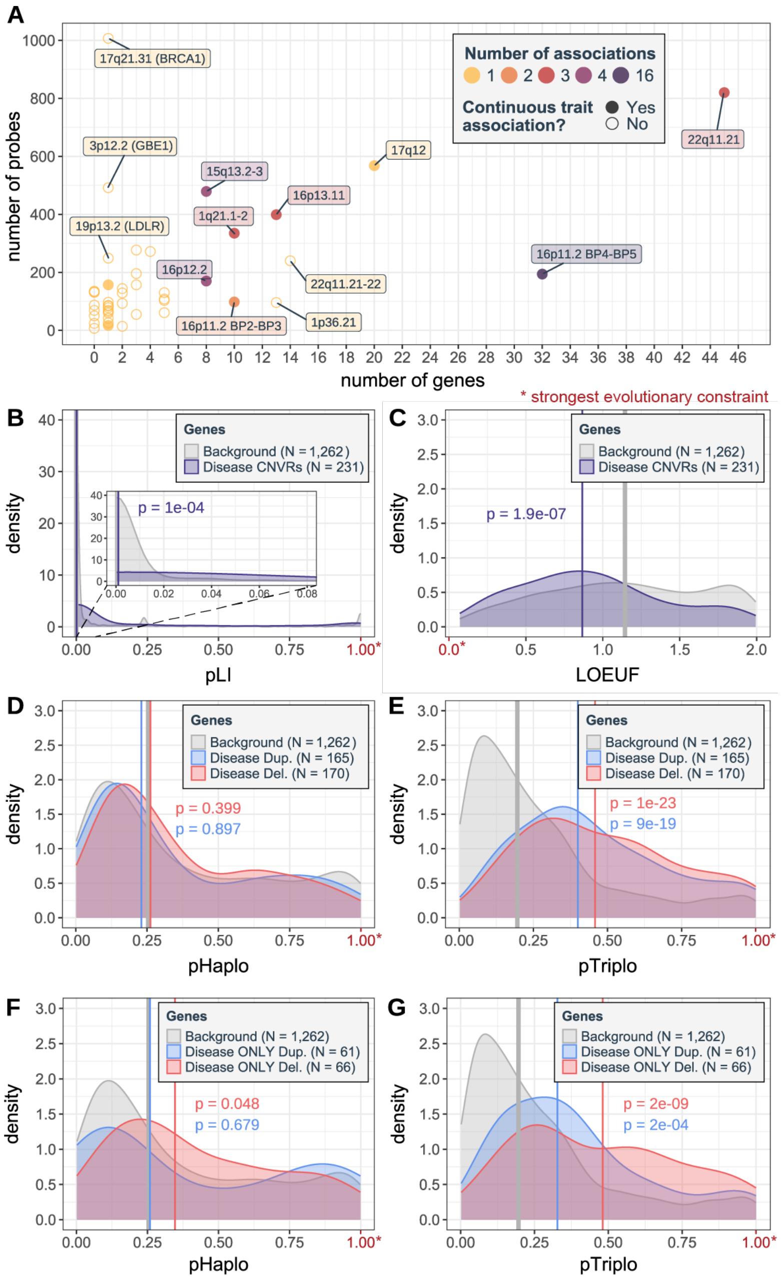

Figure S3. Constraint analysis of disease associated CNV regions

(**A**) Number of probes (y-axis) versus number of affected genes (x-axis) for disease associated CNV regions (CNVRs). Color reflects the number of associations, with full circles indicating previous association with continuous traits [1]. CNVRs affecting ≥ 6 genes or single-gene CNVR affecting > 200 probes are labeled with cytogenic bands. (**B**-**G**) Evolutionary constraint of CNVR-encompassed genes (i.e., "disease genes"): Distribution of (**B**) pLI and (**C**) LOEUF scores for disease genes versus background genes (i.e., genes overlapping regions with a CNV frequency ≥ 0.01% but no disease association). Distribution of (**D**) pHaplo and (**E**) pTriplo scores for genes overlapping CNVRs significantly associated with a disease through the duplication-only or deletion-only models versus background genes. Distribution of probability of (**F**) pHaplo and (**G**) pTriplo scores for genes overlapping CNVRs *uniquely* associated to a disease through the duplication-only or deletion-only model versus background genes. Number of genes (N) and the median score (vertical line) is indicated for each group. P-values compare groups versus background gene medians (two-sided Wilcoxon test). Direction of strongest evolutionary constraint is indicated in red with a star.

1. Auwerx C, Lepamets M, Sadler MC, Patxot M, Stojanov M, Baud D, et al. The individual and global impact of copy-number variants on complex human traits. Am J Hum Genet. 2022;109:647–68.

Figure S4: Total, corrected, and subset CNV burden analysis

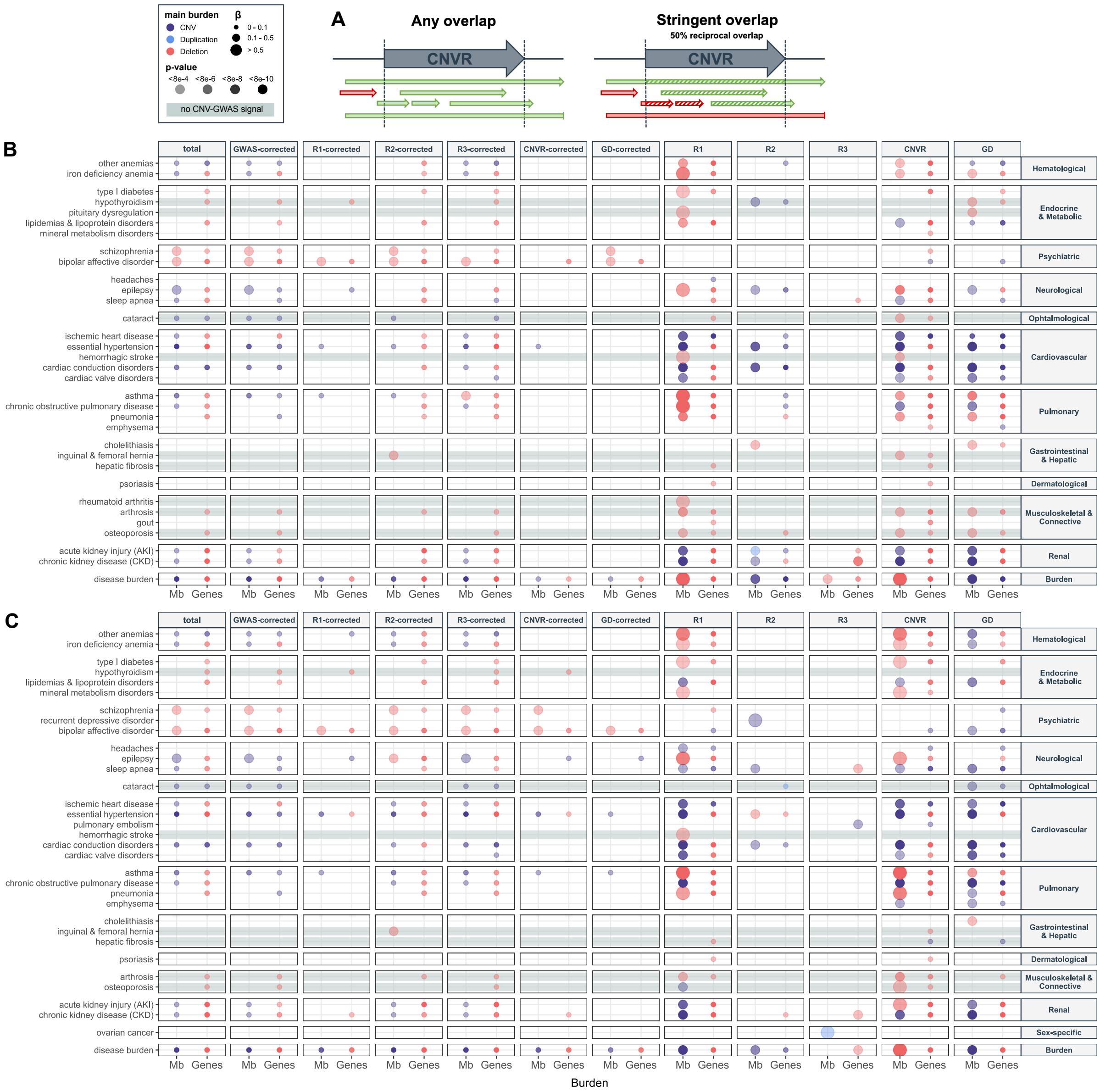

Figure S4. Total, corrected, and subset CNV burden

(**A**) Schematic representation of two ways to define overlap between a CNV and a CNV region (CNVR) in a given genomic partition. Left: Any overlap of ≥ 1 bp is sufficient to consider that the CNV overlaps the region. Right: a reciprocal 50% base pair overlap is required (stripes) to consider that the CNV overlaps the region, so that the CNV covers > 50% of the region defined by the partition and the region defined by the partition covers > 50% of the CNV. CNVs considered as overlapping are depicted in green, those that are not in red. CNVs with blunted arrows extend over a range longer than the depicted CNVR. (**B**-**C**) Contribution of the total CNV burden, the GWAS-corrected burden, as well as the total CNV burden corrected for the 5 considered genomic partitions (i.e., R1, R2, R3, CNVR, GD) and the subset burden of the same 5 genomic partitions in number of affected Mb (x-axis; left) or genes (x-axis; right) to disease risk (y-axis) using (B) any or (C) a stringent approach to define overlap, as depicted in (A). Only the most significantly associated of the CNV (purple), duplication (blue), or deletion (red) burdens, providing $p \leq 0.05/61 = 8.2 \times 10^{-4}$, is shown. Color indicates whether the CNV (duplication + deletion), duplication, or deletion burden was most significantly associated, with size and transparency being proportional to the effect size (beta) and p-value, respectively. Gray horizontal bands mark traits with no CNV-GWAS signal.
